# Advances in Wine Yeast Autolysis: Biochemical and Molecular Mechanisms, and the Release of Organic Compounds in White and Sparkling Wines—An Updated Review

**DOI:** 10.1111/1541-4337.70458

**Published:** 2026-03-28

**Authors:** José Ricardo Machado dos Santos, Aniela Pinto Kempka

**Affiliations:** ^1^ Multicentric Postgraduate Program in Biochemistry and Molecular Biology State University of Santa Catarina Lages Brazil

**Keywords:** emerging technologies, lees aging, mannoproteins, non‐*Saccharomyces* yeasts, sparkling wine, yeast autolysis

## Abstract

Wine yeasts play a central role in alcoholic fermentation and significantly contribute to the sensory attributes of wines through cellular autolysis during lees aging (*sur lie*), especially in white wines. This process releases organic compounds that alter the wine's chemical and sensory profile. In addition to traditional aging, the use of commercial yeast derivatives and emerging technologies has enabled accelerated autolysis. This review compiles recent studies on white and sparkling wines, highlighting the predominance of *Saccharomyces cerevisiae* and the growing interest in non‐Saccharomyces yeasts. Most research focuses on sparkling wines produced by the traditional method, typically involving autolysis periods ranging from 6 to 18 months, temperatures between 12°C and 18°C, and the frequent use of synthetic wine models. The volatile fraction associated with autolysis has gained attention, with diethyl succinate proposed as a key marker, despite inconsistent findings. Genes related to autophagy are being explored as molecular markers of autolytic potential, and techniques such as ATR‐FTIR and NMR spectroscopy are being used to screen yeast strains. Emerging technologies—including pulsed electric fields, high‐hydrostatic pressure, high‐pressure homogenization, ultrasound, microwaves—show promise in enhancing autolysis and improving the release of desirable compounds. However, agronomic and technological variability challenge the generalization of specific outcomes. This underscores the need to expand research into underexplored wine categories, such as sparkling wines produced by the Charmat method, long‐maceration white wines, and low‐intervention wines, in which residual yeast cells may remain at bottling and potentially influence the final sensory profile.

## 1 Introduction

The autolysis of yeast cells involves their self‐degradation through enzymatic mechanisms, including the activity of proteases, nucleases, lipases, and β‐glucanases, which induce structural changes following cell death (Le Scanff et al. 2024; Kokkinomagoulos et al. 2024; Li et al. 2020; Comuzzo et al. 2015; Liu et al. 2016). In the winemaking of white and sparkling wines, autolysis plays a crucial role, as the contact of wines with yeasts in the autolytic stage enhances sensory complexity, improving both aromatic and gustatory attributes (Cerbu, Colibaba, Luchian, et al. 2023; Alexandre and Guilloux‐Benatier 2006). Autolysis enhances colloidal stability and, in sparkling wines, contributes to bubble quality and the release of bioactive compounds (de Iseppi, Rocca, et al. 2024). These effects result from the release of organic compounds from yeast cells, including polysaccharides, lipids, peptides, and aromatic precursors such as amino acids and fatty acids (Voce et al. 2024; de Iseppi, Rocca, et al. 2024; Sawyer et al. 2021). The practice of keeping yeast biomass in contact with the wine after fermentation, known as aging on lees or *sur lie*, promotes the release of these compounds. Following the autolysis period, sparkling wines often exhibit organoleptic characteristics described as toasted, bready, or nutty notes, along with a creamy mouthfeel (Gnoinski, Close, et al. 2021). In this way, autolysis contributes to the stylistic diversity of white and sparkling wines, whose production has shown consistent growth in recent years (OIV 2023; Shanshiashvili et al. 2024).

However, the natural occurrence of autolysis in wines during the lees aging stage is a slow process (Pons‐Mercadé et al. 2022; Porras‐Agüera et al. 2020; Tabera et al. 2006). As a result, strategies and technologies aimed at accelerating autolysis in winemaking are currently under investigation (Ruipérez et al. 2022). The analysis of the metabolite profile released during autolysis provides insights into the trend, rate, and capacity of yeast to release compounds that influence aroma, flavor, mouthfeel, and foam properties (Girelli et al. 2025). The onset of autolysis may vary depending on aging conditions, such as pH, storage temperature, ethanol content, contact time, as well as the strain and population density of the yeast (Mikuš et al. 2024; Ignacia Lambert‐Royo et al. 2022; Gnoinski, Schmidt, et al. 2021; Sawyer et al. 2021). Yeast autophagy, which precedes autolysis, involves the degradation of cytoplasmic structures and contributes to cell maintenance and adaptation to nutritional stress, as well as specific conditions such as the presence of carbon dioxide in sparkling wines, high alcohol concentrations, low pH, and low storage temperatures (Metur and Klionsky 2024; Ruipérez et al. 2022; Majeed et al. 2022; Xu and Du 2022; Gnoinski, Schmidt, et al. 2021). In addition to autophagy, apoptosis, or programmed cell death, and necrosis are processes that precede autolysis in yeast (Bidiuk et al. 2021; Fahrenkrog 2015).

In addition, fragments of the cell walls of autolyzed yeasts, particularly species from the *Saccharomyces* genus, can be used for various purposes due to their adsorptive capacity. This beneficial effect is related to the presence of macromolecules, such as mannoproteins, located on the cell surface, which can adsorb volatile compounds, among others. However, the composition of the cell wall may vary depending on the yeast strain and cultivation conditions (Bakhos et al. 2021), enabling diverse research approaches. In this context, yeast derivatives such as extracts, autolysates, and inactivated dry yeast—obtained through different inactivation, enzymatic self‐digestion, extraction, and purification processes—are widely employed in the food and beverage industry. Despite their common origin, these products exhibit significant differences in composition and application (Alves et al. 2021). Inactivated dry yeast, in particular, has been used to accelerate autolysis and enhance mannoprotein release in sparkling wines, contributing to the reduction of maturation time (Barrio‐Galán et al. 2016). It is also noteworthy that the oxidative stability of white wines depends on both native grape compounds and yeast autolysis products, which enrich the wine's antioxidant metabolome (Maxe et al. 2024).

The biochemical understanding of yeast autolysis mechanisms during winemaking involves highly complex processes and constitutes a central topic that has been extensively investigated over the past two decades through various analytical approaches. Among the main research lines, particular attention has been given to how production variables influence the release of autolysis‐derived compounds, affecting both wine quality and the potential reuse of lees as a source of valuable organic compounds. This trend is evidenced by several recent studies, which include the use of emerging technologies to accelerate autolysis—such as ultrasound (US), microwaves (MWs), and pulsed electric fields (PEFs) (Chioru et al. 2024; Gnoinski, Close, et al. 2021; Gnoinski, Schmidt, et al. 2021); the identification of peptides released by yeasts during this process (de Iseppi, Rocca, et al. 2024); biochemical comparisons of yeasts during aging on lees in sparkling wines produced by the Charmat and traditional methods (Cisilotto et al. 2023); oxygen consumption during lees aging (Romanet et al. 2023; Póns‐Mercadé et al. 2021); among other key aspects discussed in this updated review.

Furthermore, a recent investigation highlighted the role of peptides with kokumi potential, associated with the modulation of taste sensations, whose release was observed during autolysis in sparkling wines (Perenzoni et al. 2024). Although still in its early stages, this topic appears promising for future research, expanding discussions on the implications of autolysis for the sensory impact of wines. In parallel, studies on the molecular basis of autolysis in wine yeasts have proven relevant for the development of biotechnological applications. Noteworthy are investigations into the role of specific genes of *Saccharomyces cerevisiae* and its mutants in autolysis and the production of aromatic compounds (Perpetuini et al. 2021), as well as proteomic analyses associated with the autophagy of these yeasts during sparkling wine production (Porras‐Agüera et al. 2020; Porras‐Agüera et al. 2019).

In this context, the objective of this literature review is to provide a comprehensive and updated synthesis of the role of autolysis in the winemaking of white and sparkling wines. The discussion focuses on the mechanisms involved in yeast autolysis and on the organic compounds generated during this process, which have attracted growing interest in the specialized literature, namely: amino acids, peptides, polysaccharides, and volatile compounds. Although yeast autolysis in wines has been extensively studied, there remains a gap regarding integrative reviews that incorporate the most recent advances and offer a consolidated overview of the current state of the field. Therefore, this review constitutes a timely and relevant contribution aimed at expanding knowledge and promoting new perspectives in the field of wine science.

## 2 Autolysis: History and Early Advances

An investigation into the origin of the term “autolysis” reveals its initial predominance in medical literature, with early records found in editorials and technical communications. A search for the isolated term autolysis on the Scopus and ScienceDirect platforms, limited to titles, abstracts, and keywords, identified the earliest reference as a note published in Lancet (1903), entitled *Autolysis in lobar and unresolved pneumonia*. Shortly thereafter, in 1905, an editorial in the *Journal of the American Medical Association* discussed the subject in the section *The proteolytic intracellular ferments and autolysis*, reporting that Salkowski, in 1890, demonstrated the ability of hepatic and muscular enzymes to degrade albumins, forming amino acids such as leucine and tyrosine. Initially described as “self‐digestion,” this process came to be widely referred to as “autolysis,” a term proposed by Jacoby (JAMA 1905).

Even earlier, Salkowski (1889) had already reported the production of organic acids, amino acids, and carbohydrates during yeast autolysis. In 1919, a new editorial in the *Journal of the American Medical Association* revisited the topic, reiterating that the term “autolysis” was introduced by Jacoby in 1900 and highlighting its growing role in chemical pathology research (JAMA 1919), a point also emphasized by Dernby (1918). In parallel, Levene (1906) discussed autolysis in relation to grape alcoholic fermentation, based on Eduard Buchner's discoveries regarding extracellular enzyme‐mediated fermentation, for which he was awarded the Nobel Prize in Chemistry in 1907 (Jaenicke 2007). Levene emphasized that this discovery “marked a radical shift in our conception of the life process.” He also attributed to Salkowski the observation that dead cells are still capable of undergoing self‐disintegration, while acknowledging earlier contributions by Hoppe‐Seyler in 1871. Levene (1906) noted that Hoppe‐Seyler's observations (1871) demonstrated that, even in the absence of oxygen, dead tissues underwent a softening and dissolution process similar to putrefaction, though without a fetid odor, resulting in the formation of compounds such as leucine, tyrosine, acids, and soaps. This phenomenon was interpreted as a form of enzymatic self‐digestion independent of external microorganisms. In the same text, Levene also mentioned Schützenberger's experiments from 1874, who observed similar changes in yeasts maintained in aqueous suspension for 12–15 h at temperatures between 35°C and 40°C, suggesting that such self‐digestion processes also occurred in unicellular organisms such as yeasts.

In his study, Dernby (1918) cites the English botanist Vines (1904, 1909), who proposed the existence of two distinct proteolytic enzymes in yeast cells: one similar to pepsin and another to trypsin. Previous studies had already identified proteolytic activity in yeasts, including the works of Geret and Hahn in 1898 and Cohnheim in 1901, as noted by Vostin and Joslyn (1954). Dernby proposed the existence of three groups of proteolytic enzymes in yeasts: peptic, tryptic, and ereptic in nature. These pioneering studies demonstrate that, although the initial focus was directed toward animal cells and tissues, yeasts were also among the earliest biological models used in the study of autolysis. Among the most notable examples are the works of Navassart (1910, 1911), who investigated the influence of pH and antiseptic agents on yeast autolysis.

**FIGURE 1:**
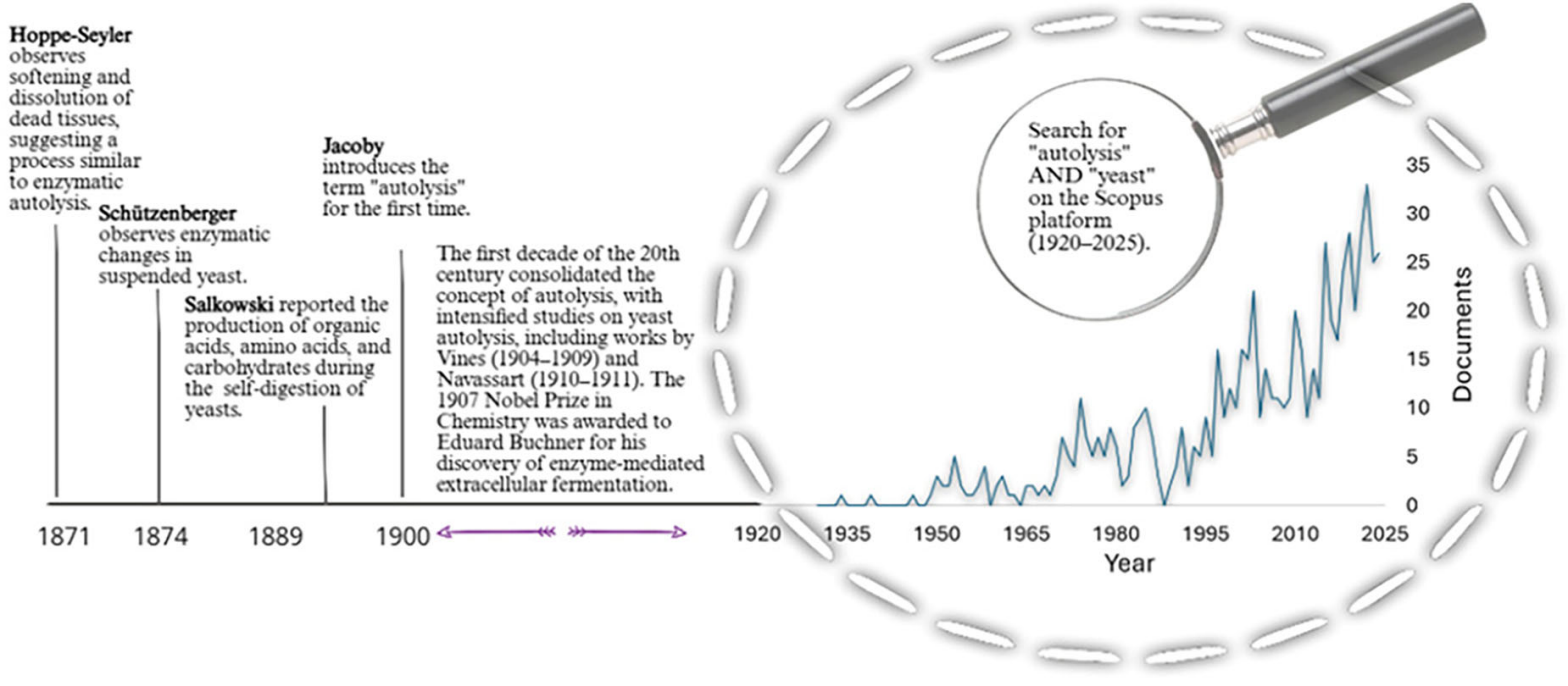
Timeline of key events related to the earliest mentions of autolysis, based on the reviewed documents and the results of a search conducted on the Scopus platform for the period from 1920 to 2025.

In 1922, Shin Shima published the article *Studies of Autolysis*, in which he emphasized that fundamental questions regarding the nature of the enzymes involved in autolysis and the influence of various agents remained unanswered (Shima 1922). This scenario underscores the intense scientific interest devoted to the topic during the early decades of the 20th century. Three decades later, Vostin and Joslyn (1954) published a study on autolysis in baker's yeast, highlighting the growing interest of the baking, brewing, and winemaking industries, which significantly boosted research on yeast autolysis. This trend is corroborated by a literature survey conducted on the Scopus platform, which shows a progressive increase in publications containing the Boolean operators “autolysis” AND “yeast” in titles, abstracts, and keywords since the 1950s, as illustrated in Figure 1.

## 3 Aging on Lees and Autolysis in White Wines

This section describes the stages involved in lees aging and yeast autolysis during the production of still and sparkling white wines. The differences between winemaking methods and their implications for the biochemical and sensory processes associated with autolysis are discussed.

### 3.1 Still White Wines

In the case of still white wines, the process begins with the extraction of the grape must, which undergoes pre‐fermentative steps such as clarification and nutrient addition. At this stage, yeasts are inoculated to initiate alcoholic fermentation, unless spontaneous fermentation is chosen. At the end of fermentation, the yeast cells settle at the bottom of the vessel, allowing for possible contact of the wine with the lees (*sur lie*), which can potentially influence the wine's sensory profile. This contact may occur in stainless steel tanks or wooden barrels, resulting in different characteristics, since the micro‐oxygenation promoted by the porous structure of the wood barrels can enhance the flavor and structure of the wine, contribute to the integration of aromas, promote balance and harmony, and consequently improve the overall sensory quality of the product (Zhang et al. 2024).

**FIGURE 2:**
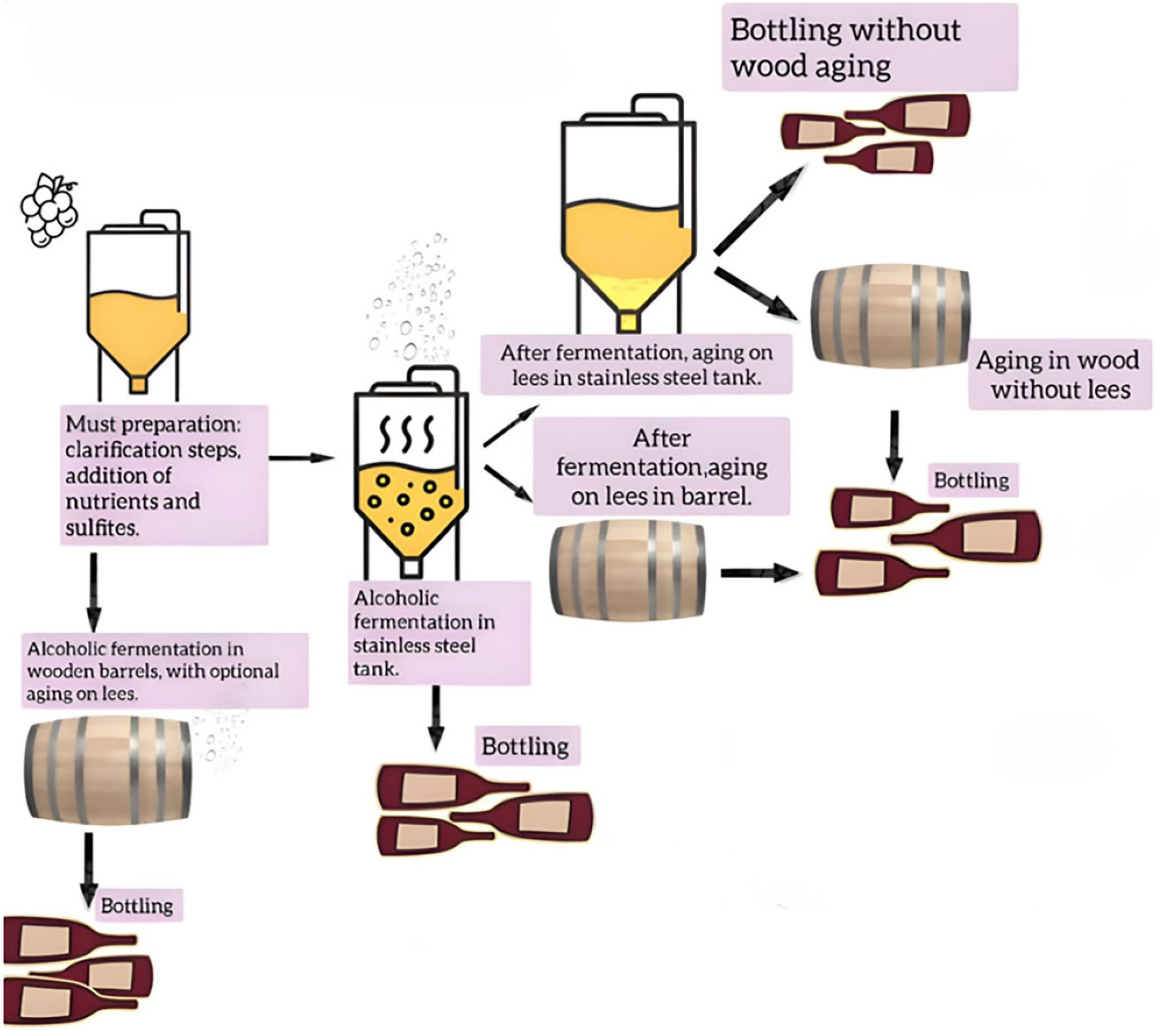
Illustrative flowchart of the still white wine production process.

Another approach involves conducting fermentation directly in barrels, which enhances the integration between wood and lees. In such cases, *bâtonnage* may be applied—a technique that involves the periodic resuspension of the lees to increase the extraction of compounds (Alexandre 2022; Zamora 2022). Regardless of the vessel type, steps such as tartaric stabilization and filtration may be carried out prior to bottling. Tartaric stabilization, typically performed under cold conditions, induces the formation of potassium bitartrate salts, thereby preventing the appearance of crystals in the bottled wine (Dabare et al. 2023). The flowchart shown in Figure 2 provides an illustration of the production process of still white wine.

### 3.2 Sparkling Wines—Charmat and Traditional Methods

The production of sparkling wines begins with the preparation of the still base wine, which is then subjected to the prise de mousse using either the Charmat or traditional method. In the Charmat method, the second fermentation takes place in pressurized tanks, following the addition of sugar and active yeasts (*liqueur de tirage*) (Perpetuini et al. 2016). Upon completion of fermentation, the lees settle at the bottom of the tank, initiating a potential aging period on lees, which may or may not be employed depending on the desired wine style.

The main difference between the methods lies in how the second fermentation and subsequent steps are conducted. In the traditional method, fermentation takes place directly in the bottle, which is stored in a horizontal position, promoting more continuous and localized interaction with the lees. The smaller scale of the bottle also results in a more favorable surface‐to‐volume ratio, contributing to greater sensory complexity. In contrast, in the Charmat method—particularly when tanks equipped with agitators are used—it is possible to keep the wine homogenized during both fermentation and aging, with the lees remaining in suspension. This condition increases the contact area between the yeasts and the wine, enhancing the release of autolytic compounds (Cisilotto et al. 2023). The study by Cisilotto et al. (2023) compared the effects of continuous agitation in the Charmat method with the static system characteristic of the Traditional method. The authors observed that homogenization in the Charmat method leads to a faster decline in cell viability, accompanied by an increase in the expression of genes related to autophagy, suggesting early activation of this process. These findings indicate functional differences between suspended and sedimented cells, although the authors emphasize that the final sensory outcomes may be similar in both methods.

**FIGURE 3:**
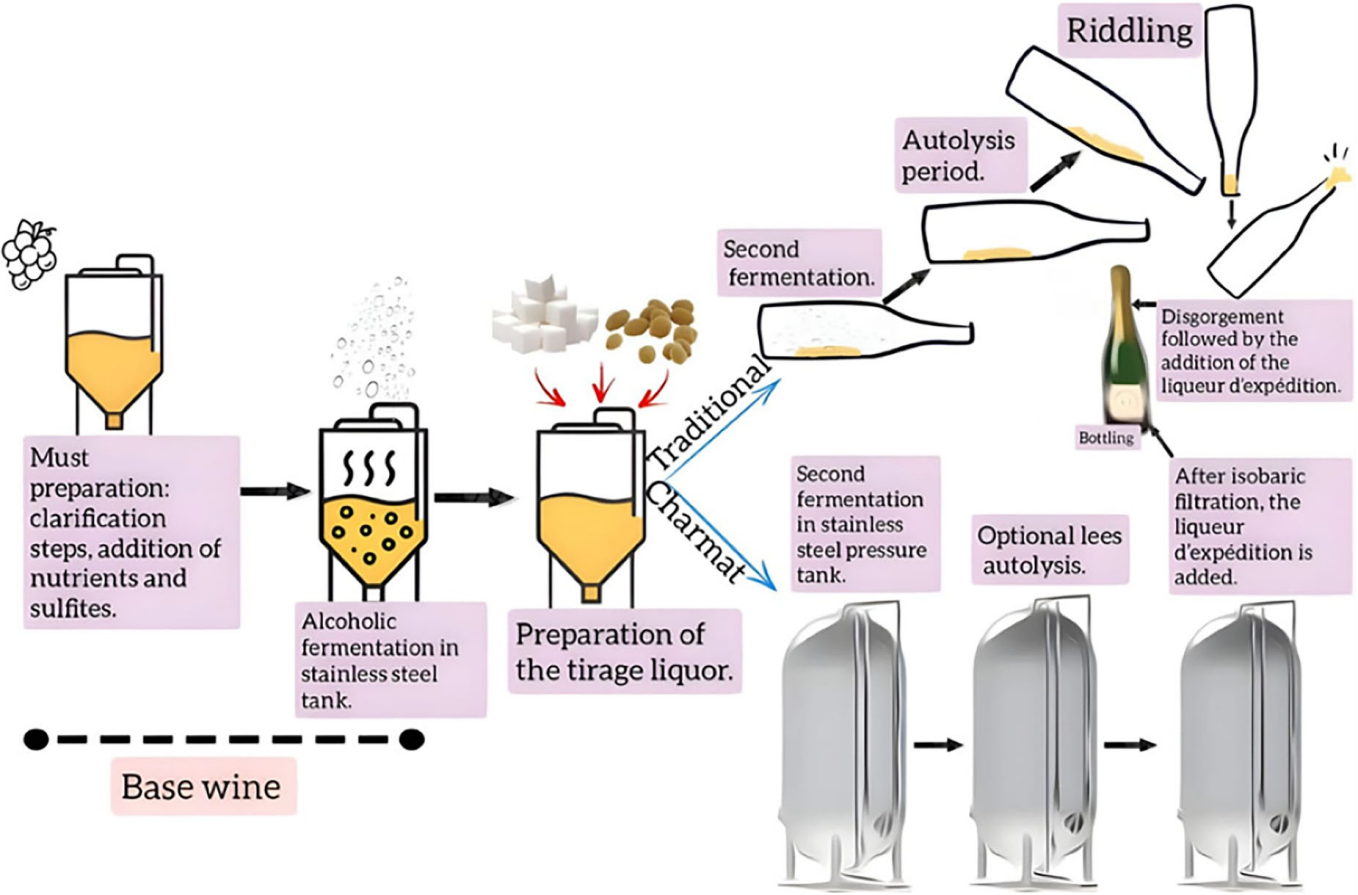
Illustrative flowchart of the sparkling white wine production process.

At the end of the aging process, the sparkling wine is separated from the lees and may undergo filtration prior to bottling. In the traditional method, following in‐bottle fermentation and yeast autolysis under pressure ranging from five to six atmospheres (Taranenko et al. 2023; Perpetuini et al. 2016), the *remuage* is carried out—a process involving the gradual rotation and inclination of the bottles to concentrate the sediment in the neck, which can be performed manually or mechanically. Next comes the *dégorgement*, during which the neck of the bottle is frozen and the cap removed, allowing the lees to be expelled. Finally, the *liqueur d'expédition* is added to adjust sweetness, and the bottle is sealed with a cork, completing the production process. The flowchart shown in Figure 3 provides an illustration of the production process of sparkling white wine.

## 4 Biochemical and Molecular Bases of Autolysis in Wine Yeasts

This section explores the biochemical and molecular foundations underlying autolysis in wine yeasts. Structural features, metabolic pathways, regulatory genes, and cellular mechanisms responsible for the release of compounds during lees aging are addressed, with a particular focus on S. cerevisiae and its technological applications.

Yeasts are unicellular eukaryotic organisms composed of a cell wall, plasma membrane, cytoplasm, and nucleus (Togores 2018). Enzymes produced by the yeast cell itself initiate the autolysis process, such as proteases, which in *S. cerevisiae* are released from vacuoles and hydrolyze cytoplasmic proteins (Li et al. 2020). Proteases A (PrA) and B (PrB) account for 80%–90% of vacuolar proteolytic activity (Dimopoulos et al. 2021), leading to the release of compounds such as fatty acids, peptides, amino acids, and nucleotides (Cerbu, Colibaba, Luchian, et al. 2023). During active cell life, these vacuolar proteases are functionally inhibited by specific cytoplasmic inhibitors. Upon the onset of autolysis, these inhibitors are degraded, allowing intracellular proteolytic activation. The hydrolysis of cellular components begins, and the hydrolyzed compounds are released either when their molecular weight becomes sufficiently low or when the cell wall and membrane are degraded (Pons‐Mercadé et al. 2022; Alexandre 2011). Due to the low enzymatic activity under typical white wine aging‐on‐lees conditions—characterized by low pH and temperatures generally below 15°C—autolysis progresses slowly (Mikuš et al. 2024), requiring prolonged contact with the lees, often lasting several months or even years, for its beneficial effects to be fully achieved (Tofalo et al. 2022).

The enzymatic degradation of the yeast cell wall leads to the release of various compounds of interest, whose composition depends on the yeast species used (Jofre et al. 2024). The yeast cell wall accounts for approximately 15%–30% of the cell's dry weight and is composed of components of high value for both the food industry and the wine lees aging process, particularly mannoproteins and β‐glucans (Berzosa et al. 2023). Of these, around 35%–40% of the wall's dry weight consists of mannoproteins—glycosylated proteins located in the outermost layer—while 50%–55% consists of β‐glucans, glucose polymers found in the inner layer that confer structural rigidity (Berzosa et al. 2023). These β‐glucans form an interlaced network with chitin, composed mainly of long β‐1,3‐glucan chains with branches linked by β‐1,6 bonds. β‐1,3‐Glucanase enzymes act on this network by hydrolyzing β‐1,3 linkages, facilitating the disassembly of the cell wall structure (Bartolo‐Aguilar et al. 2017). β‐Glucanases are of particular interest in the wine industry due to their ability to degrade the cell wall, promoting the release of mannoproteins and oligosaccharides during lees aging, as well as aiding in the filtration of musts and wines made from grapes affected by *Botrytis cinerea* (Schwentke et al. 2014).

The regulation of yeast cell wall biosynthesis involves both transcriptional and posttranscriptional control mechanisms, coordinating gene expression and the synthesis of enzymes and polysaccharides in a temporally and spatially regulated manner (Penacho et al. 2012). The formation of the polysaccharides chitin and β‐1,3‐glucan occurs at the plasma membrane through transmembrane enzymatic complexes, using UDP‐glucose and UDP‐*N*‐acetylglucosamine as precursors. In contrast, the synthesis of mannans and β‐1,6‐glucans involves proteins encoded by specific genes located in the endoplasmic reticulum and Golgi apparatus, with mannans being subsequently transported to the cell wall (Jofre et al. 2024). Gow et al. (2017) report that approximately one‐fifth of the yeast genome is dedicated to cell wall biosynthesis, with around 1200 genes in *S. cerevisiae* involved in its construction and maintenance. Non‐*Saccharomyces* yeasts, which have garnered increasing attention in the enological context, are capable of synthesizing larger amounts of polysaccharides during alcoholic fermentation compared to *S. cerevisiae*. This may be related to a higher production capacity or to faster cell renewal during the multiplication phase. This characteristic is particularly relevant when considering the potential to shorten lees aging time through the use of strategic yeast strains (Voce et al. 2022).

Regarding the molecular mechanisms involved in autolysis, the deletion of *FLO1* and *FLO5* genes in *S. cerevisiae* resulted in distinct autolytic behaviors, with the mutants exhibiting increased autolysis, as evidenced by greater amino acid release—particularly in the non‐flocculent mutant used in the second fermentation of sparkling wines. The *FLO1* and *FLO5* genes may exert pleiotropic effects on yeast phenotypes, including autolytic capacity, and it is likely that flocculation plays a significant role in modulating this process (Perpetuini et al. 2021). Indeed, *FLO1*, *FLO5*, *FLO9*, *FLO10*, and *FLO11* are identified as key genes responsible for flocculation in *S. cerevisiae* (di Gianvito, Tesnière, et al. 2018). Flocculation, resulting from the phenotypic expression of these genes, is viewed as a form of social cooperation that protects the cells within the floc from various stressors. This process also promotes the compaction and sedimentation of yeast biomass, aiding in its removal during the *dégorgement* step of sparkling wine production via the Traditional method (Di Gianvito et al. 2018).

In the search for genetically modified wine strains of *S. cerevisiae*, Tabera et al. (2006) investigated how partial or total deletions of the *BCY1* gene affect autolysis, as mutations in this gene may lead to reduced cell viability in response to nutrient limitation. Heterozygous strains carrying these deletions exhibited a semidominant phenotype for autolysis under second fermentation conditions. The selection of mutants with modifications in *BCY1* is justified by their tendency to lose viability soon after glucose depletion, allowing fermentation to be completed. Based on these findings, the authors proposed that deletion of the 3′ end of a single *BCY1* copy improves the quality of sparkling wines. This gene encodes the regulatory subunit of the cAMP‐dependent protein kinase A (PKA), a signaling pathway activated by various environmental cues and crucial for the control of metabolism and stress response (Tabera et al. 2006). Another pathway associated with autolysis in baker's *S. cerevisiae* strains is the mitogen‐activated protein kinase (MAPK) pathway, which regulates the cell cycle and stress‐induced cell death. Key genes involved in this pathway include *MID2*, *MTL1*, *SLT2*, *PTP2*, *HKR1*, and *GPD*, all of which can be activated by extracellular signals such as cytokines and hormones, suggesting their role in the molecular mechanisms of yeast autolysis (Li et al. 2020).

Autophagy‐related genes are considered promising candidates for investigating the molecular basis of autolysis and for applications in the genetic engineering of wine yeasts (Perpetuini et al. 2016). The overexpression of *ATG3* and *ATG4*, both associated with autophagy, was studied in *S. cerevisiae* to evaluate their impact on cellular homeostasis under nitrogen starvation and fermentation efficiency during the refermentation of base wine for sparkling wine production (Preiss et al. 2018). Genetic manipulation accelerated cell death in different yeast strains using two distinct promoter replacement systems (*CUP1* or *PGK1*). According to the authors, these findings suggest that increased expression of *ATG* genes may intensify autolysis, with potential implications for the sensory quality of sparkling wines. Autophagy‐related genes *ATG1*, *ATG17*, and *ATG29* were also examined in another study focusing on autolysis in 12 *S. cerevisiae* strains used in sparkling wine production (Perpetuini et al. 2016). Porras‐Agüera et al. (2020) investigated the relationship between autophagy and autolysis in *S. cerevisiae*, aiming to enhance the organoleptic properties of sparkling wines. Two industrial strains were used: P29 (applied in sparkling wine production) and G1 (associated with flor yeast and Sherry wine production). CO_2_ pressure and secondary fermentation affected the autophagy‐related proteome, with more pronounced effects observed in the G1 strain. Proteins such as Bcy1p, Sec2p, Sec13p, Sec18p, Shp1p, and Vps15p were detected in higher concentrations under pressure, potentially serving as biomarkers to accelerate autolysis during aging. In the G1 strain, regulatory proteins and those involved in autophagosome formation were prominent, while in P29, proteins associated with vesicle nucleation and expansion were more relevant. These findings suggest that autophagy can be strategically modulated to optimize autolysis and enhance the sensory quality of sparkling wines.

Among the limited studies comparing the Charmat and traditional methods, Cisilotto et al. (2023) observed an earlier and more intense expression of genes related to macroautophagy—such as *ATG8* (involved in autophagosome formation), *AMS1* (mannosidase), and *APE1* (vacuolar aminopeptidase)—in the Charmat method compared to the traditional one. In contrast, genes associated with apoptosis—*YCA1*, *AIF1A*, and *NUC1*—showed low expression in both methods, suggesting a limited role of this pathway in cell viability loss. The authors propose that the greater induction of autophagy in the Charmat method is linked to the continuous homogenization that keeps cells in suspension, unlike the static fermentation of the traditional method, where yeasts settle at the bottom of the bottles. Therefore, the hypothesis raised is that the physical environment in the Charmat method promotes cellular stress and autophagic activation, contributing to a more pronounced reduction in yeast viability in this system.

The generation of autolytic mutants through UV mutagenesis was employed to investigate the behavior of different *S. cerevisiae* strains during the refermentation of base sparkling wine from the Parellada variety (Nunez et al. 2005). Among the strains evaluated, the mutant IFI473I exhibited accelerated release of proteins, amino acids, and polysaccharides during aging. Mannose was identified as the main sugar released, contributing to the composition of the sparkling wine. Wines fermented with the IFI473I strain showed improved foam formation compared to those fermented without it, an effect observed even with a shortened aging period of 6 months. These findings highlight the strain's capacity to promote accelerated autolysis and enhance the sensory characteristics of sparkling wines. More recently, the novel application of ^1^H NMR spectroscopy has been explored as a promising approach for selecting *S. cerevisiae* starter cultures intended for the second fermentation of sparkling wines. This technique demonstrated potential as an analytical tool for monitoring autolysis‐associated markers, thereby aiding in the selection of strains with optimized enological performance (Girelli et al. 2025).

Moreover, the role of *S. cerevisiae* strains possessing the *killer* factor, which may influence autolysis, is noteworthy. Strains with phenotypic characteristics of *killer* factors, as well as sensitive strains, have been identified as having potential to accelerate cell death during autolysis, thereby enhancing foam formation and the organoleptic quality of sparkling wines undergoing second fermentation in the bottle, particularly when cultured in mixed populations of *killer* and sensitive strains (Velázquez et al. 2016). In selecting *S. cerevisiae* strains for the second fermentation of sparkling wines produced by the traditional method, specific phenotypic traits are prioritized—such as high autolytic capacity and strong flocculation—which are less critical in strains intended for still wine production (di Gianvito, Perpetuini, et al. 2018). Furthermore, the conditions of the base wine used for refermentation differ significantly from those of the grape must used for still or base sparkling wines. Therefore, in addition to flocculation and autolysis, selected strains must demonstrate resilience to multiple stress factors, including high ethanol concentrations, low fermentation and aging temperatures, reduced pH, high total acidity, nutrient limitation, and the pressure generated by CO_2_ (di Gianvito, Perpetuini, et al. 2018).

Still within the scope of sparkling wines, Cebollero et al. (2009) employed genetic engineering techniques to develop an industrial autolytic strain by expressing the *csc1‐1* allele at the *RDN1* locus. To achieve this, a strain of *Escherichia coli* was used for the construction and amplification of the necessary plasmids. As in the other studies mentioned, the commercial strain used for the second fermentation belonged to the species *S. cerevisiae*. The integration of the *csc1‐1* allele—associated with autophagy—into the yeast genome aimed to accelerate the autolysis process during sparkling wine production. The combination of accelerated autolysis with the preservation of fermentative capacity demonstrated that *csc1‐1* overexpression is a viable strategy for producing wines with characteristics similar to traditionally aged products in a shorter time frame. In a separate study, Penacho et al. (2012) evaluated the recombinant *S. cerevisiae* strain EKD‐13, which carries a deletion in the *KNR4* gene. This modification promoted the release of mannoproteins during fermentation, even with minimal aging on lees in synthetic must. Gene expression analysis revealed that *KNR4* deletion affected processes such as flocculation, anaerobic adaptation, and oxidative stress response, while also enhancing ethanol tolerance. Compared to the parental strain, EKD‐13 released higher amounts of mannoproteins by the end of fermentation without requiring extended aging. However, the authors suggest that aging wine on EKD‐13 lees could yield additional sensory benefits due to the release of intracellular components.

In the context of emerging technologies, which are addressed in Section 6, high‐pressure homogenization (HPH) has been investigated for its effects on gene expression, cell cultivability, and volatile compound profiles in *S. cerevisiae* grown in synthetic must (Gottardi et al. 2022). Although yeast viability and cultivability were not compromised, the treatment induced differential expression of 1220 genes. Genes related to energy metabolic pathways and ribosomal structure were downregulated, while genes involved in ribosome maturation, transcription, DNA repair, and stress response were upregulated, suggesting the induction of an autolysis‐like state. In addition, the treatment altered the volatile compound profile. Another study, using *Saccharomyces bayanus*, showed that HPH also triggered gene expression changes, particularly in *OLE1*, *ERG3*, and *ERG11*, which are involved in the synthesis of unsaturated fatty acids and sterols. The addition of exogenous unsaturated fatty acids to the growth medium suppressed the expression of *OLE1* and *ERG3* and altered the membrane lipid composition, suggesting a role for these lipids in the cellular response to mechanical stress induced by HPH (Serrazanetti et al. 2015). These findings support the hypothesis that this technology induces genetic reprogramming in yeasts, leading to a physiological state compatible with autolysis, as observed in *S. cerevisiae* (Gottardi et al. 2022).

The studies mentioned demonstrate that recombinant strains and proteomic analyses have been extensively applied to *S. cerevisiae* to investigate the molecular and physiological impacts on autolysis. This species is favored due to its high tolerance to alcoholic stress, particularly relevant in the second fermentation of sparkling wines, which occurs in an ethanol‐rich environment. *S. cerevisiae* comprises various commercial strains selected for their physiological and technological characteristics, including fermentative capacity, flocculation, pronounced autolytic properties, and contribution to wine foaming (la Gatta et al. 2016). Most research in the field of autolysis and aging on lees has focused on the application of *S. cerevisiae* in sparkling wines, whereas other yeast species remain underexplored (Voce et al. 2024). Findings by Voce et al. (2022) show that *Hanseniaspora* spp. is capable of producing and releasing polysaccharides, amino acids, and antioxidant compounds during its growth and autolysis, with biomass and cell viability levels comparable to those of commercial active dry yeasts. These results highlight the importance of understanding the complex biochemical and molecular mechanisms across different yeast species, particularly regarding their suitability for technological applications in the wine industry. Furthermore, other non‐*Saccharomyces* yeasts mentioned by Voce et al. (2024)—including *Torulaspora* spp., *Lachancea* spp., *Metschnikowia* spp., and *Schizosaccharomyces* spp.—are identified as promising candidates for future research. These species show potential for innovative uses of yeast lees as auxiliary agents in winemaking, laying the foundation for an integrated and forward‐looking approach aligned with the principles of sustainable enology.

**FIGURE 4:**
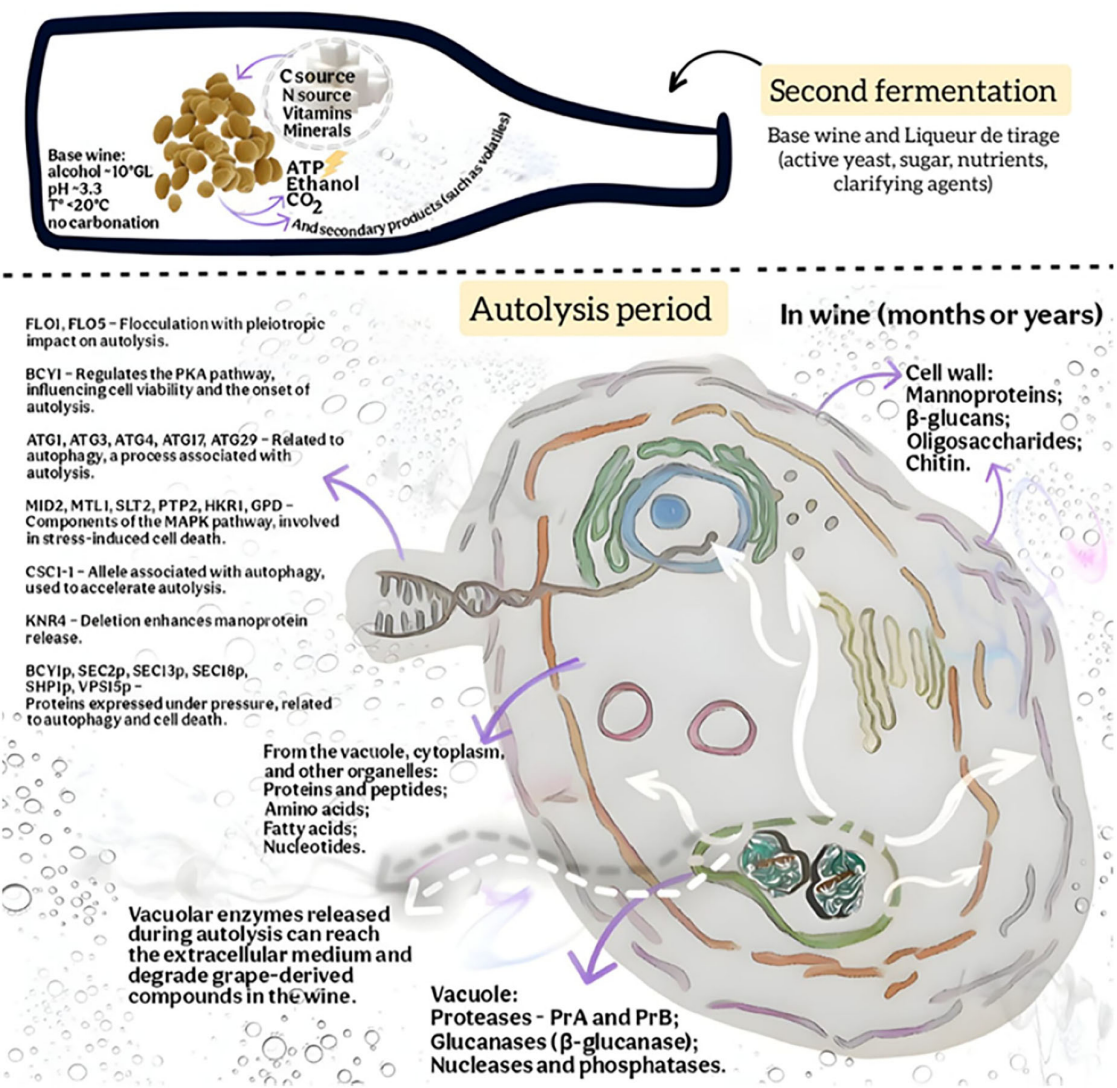
Illustration of the fermentative dynamics during the prise de mousse using the traditional method.

Figure 4 illustrates the key events that take place during the second fermentation of sparkling wines produced by the traditional method. The diagram also highlights molecular biology findings related to autolysis, including the release of compounds by yeast cells and by the various structures that make up their cell wall and membrane.

## 5 Dynamics of Organic Compounds During Yeast Autolysis and Lees Aging

This section explores the main groups of compounds released during the autolysis process, with a focus on amino acids, peptides, polysaccharides, and volatile compounds, as well as other related elements discussed in association with these groups. Emphasis is placed on their technological and sensory functions in the evolution and quality of wines during lees aging.

### 5.1 Amino Acids

Amino acids are utilized by yeasts for cell multiplication and other vital functions, with their uptake mediated by amino acid‐polyamine‐organocation (APC) transporters, which exhibit varying affinities for extracellular substrates (Kawano‐Kawada et al. 2018). As previously mentioned, vacuoles and lysosomes—organelles containing hydrolytic enzymes active at acidic pH—are responsible for the degradation of macromolecules such as proteins, nucleic acids, and lipids, particularly under nutrient‐limited conditions, when autophagy and subsequent degradation of intracellular organelles and cytoplasmic proteins occur (Kawano‐Kawada et al. 2018). Along with inorganic ammonium salts, amino acids constitute the yeast‐assimilable nitrogen (YAN) fraction, playing a crucial role in the kinetics of grape must fermentation. On average, yeasts require approximately 140 mg/L of assimilable nitrogen to meet their metabolic demands, including protein synthesis and biomass production necessary for completing fermentation (Onetto et al. 2024). To meet this nitrogen requirement, organic sources such as yeast extract and peptone are commonly used. Yeast extract, in particular, is a highly nutritious powder obtained primarily from *Saccharomyces* yeasts cultivated for this purpose. It is notable for its high content of amino acids, peptides, purines, pyrimidines, B‐complex vitamins, and minerals, which support yeast growth and metabolic activity. Peptone, derived from protein hydrolysis, is also widely used as a source of assimilable nitrogen. However, both sources are costly, potentially accounting for 30%–40% of expenses in fermentation processes due to the complex production methods involved, such as autolysis, plasmolysis, and mechanical lysis (Kokkinomagoulos and Kandylis 2024).

The signaling endoprotease Ssy5 is a key component of the SPS sensing pathway in *S. cerevisiae*, enabling cells to detect extracellular amino acids and initiate their uptake. The binding of amino acids to the SPS sensor triggers the endoproteolytic cleavage of the transcription factors Stp1 and Stp2. Once their N‐terminal domains are cleaved by Ssy5, these factors translocate to the nucleus and induce the expression of genes encoding amino acid permeases (Martins et al. 2018). Conversely, it has been observed that in live *S. cerevisiae* cells, under conditions of amino acid overproduction or impaired uptake, amino acid release can occur via the Aqr1 transporter, encoded by the *YNL065w*/*AQR1* gene. This transporter mediates the transport of amino acids such as threonine and homoserine into internal vesicles, which subsequently release them into the extracellular environment via exocytosis (Velasco et al. 2004). Furthermore, *AQR1* overexpression was found to increase the excretion of amino acids such as alanine, aspartate, and glutamate, which are relatively abundant in the cytosol (Velasco et al. 2004).

In the study by Martí‐Raga et al. (2016), it was observed that the release of the nitrogenous fraction began relatively early after the completion of the second fermentation in sparkling wines. This phenomenon was described by the authors as cellular excretion and should not be mistaken for compound release resulting from autolysis. The excreted amino acids can also undergo decarboxylation, deamination, and synthesis reactions, leading to the formation of aromatic compounds characteristic of sparkling wines. It is reported that sulfur‐rich amino acids such as cysteine and methionine can give rise to toasted nut and coffee aromas, and also participate in reactions with lipids, contributing to the development of new flavors and aromas (Prokes et al. 2022). This process is directly associated with the release of intra‐ and extracellular proteases that degrade proteins into peptides, which are subsequently converted into free amino acids (Sirisena et al. 2024; Perpetuini et al. 2016). In this context, it is important to consider the origin of the degraded proteins—whether from the grapes or the yeasts—since yeast‐derived proteases can also act on grape proteins. Therefore, the dynamics of this proteolytic conversion depend on the initial protein concentration in the must, the clarification processes (which may remove these proteins), and the yeast strain used, as proteolytic activity varies among different strains (Martí‐Raga et al. 2016). In this regard, the use of synthetic wines is particularly valuable, as it enables the isolation of the effects of the microorganisms of interest without interference from similar grape‐derived compounds.

The transformation of proteins into amino acids gives yeast biomass high biological value, as yeast proteins contain all essential amino acids (Berzosa et al. 2023). Endogenous yeast proteases exhibit specificity for particular cleavage sites, and the quantity of these enzymes varies according to the physiological state of the cells (Sirisena et al. 2024). In addition, endogenous nucleases can degrade DNA and RNA macromolecules into smaller fragments (Kokkinomagoulos et al. 2024). During the aging of sparkling wines by the Traditional method, Martínez‐Rodríguez et al. (2002) described four distinct stages of modification in nitrogen composition. In the first stage (0–40 days), the second fermentation occurs, with a decrease in amino acids and proteins and the release of peptides. In the second stage (40–90 days), viable and dead cells coexist, leading to the release of nitrogen compounds and increased proteolytic degradation. In the third stage (90–270 days), viable cells disappear, and the release of proteins and peptides predominates. Finally, in the fourth stage (after 270 days), a reduction in amino acid levels is observed in some wines.

With regard to amino acids, biogenic amines are also relevant, as they are formed from amino acid precursors during wine fermentation, maturation, aging, and storage, with the involvement of microorganisms (Cerbu, Colibaba, Popîrdă, et al. 2023). The formation of biogenic amines was evaluated in still white wines over a 12‐month autolysis period using Sauvignon Blanc and Busuioacă de Bohotin grapes (Cerbu, Colibaba, Popîrdă, et al. 2023). The quantified amines included ethanolamine, phenylethylamine, histamine, tyramine, spermidine, cadaverine, and putrescine. The slight increase in these amines was attributed to hydrolysis and decarboxylation processes of nitrogenous compounds during proteolysis. Among the precursors, l‐serine—decarboxylated to form ethanolamine—was noted, as well as l‐tyrosine, which leads to tyramine, and l‐arginine, which gives rise to putrescine (Cerbu, Colibaba, Popîrdă, et al. 2023). In a separate study, total biogenic amine production was found to be higher in rosé sparkling wines compared to white sparkling wines, which corresponded to higher amino acid concentrations in the rosé samples. Nevertheless, the detected levels were well below toxic thresholds (Martínez‐Lapuente et al. 2018).

The role of amino acids in foam quality is also significant: at wine pH, when protonated, they act as cationic surfactants, with their hydrophobic side chains favoring accumulation at liquid–gas interfaces, thereby enhancing the foamability of sparkling wines. Among the amino acids analyzed, β‐alanine and glutamic acid showed the strongest positive correlations with the maximum foam height reached (Martínez‐Lapuente et al. 2015). It is important to note that although amino acid release through autolysis provides sensory benefits to wines, excessive concentrations may compromise microbiological stability during lees aging, particularly when SO_2_ or other antimicrobial levels are insufficient (Voce et al. 2022).

An additional approach to amino acids concerns their impact on taste perceptions. In this context, umami flavor is frequently cited, with monosodium glutamate being the primary compound responsible for this taste (Franceschi et al. 2023). Furthermore, agronomic factors significantly influence the amino acid fraction in wines. Culbert et al. (2017) note that amino acid composition can vary depending on vineyard management, water availability, and nitrogen application, with proline accumulation being a physiological response to stress. Variability was also observed among wines produced by the same method: in carbonated wines, amino acid levels ranged from 471 to 1924 mg/L, with differences attributed to grape variety and/or vineyard practices (Culbert et al. 2017). Regarding proline, Girelli et al. (2025) report that although it is not used by yeasts as a nitrogen source, it plays a critical role as a cellular protector against abiotic stresses, such as ethanol. Its cytosolic levels are regulated by exchanges with the vacuole, and variations in its concentration among samples of the same grape variety may indicate different stages of cellular autolysis, with lower levels suggesting later release of this metabolite. Unlike proline, alanine is actively involved in cellular metabolism and its concentration depends on the yeast strain. Derived from pyruvic acid, alanine can be formed through decarboxylation of aspartic acid, transamination, or through the action of ammoniacal nitrogen. Low levels of alanine in certain sparkling wines may indicate subsequent transformations such as decarboxylation, deamination, or synthesis. In addition, alanine contributes to the Maillard reaction during sparkling wine aging (Girelli et al. 2025). Table 1 presents studies published between 2015 and 2024 reporting relevant findings on the impact of autolysis on the release of amino acids in wines.

**TABLE 1:** Studies published between 2015 and 2024 on yeast autolysis and its effects on amino acid release in wines.

References	Grape varieties	Wine type	Lees aging conditions (autolysis) and other relevant parameters	Implications for amino acids
Mikuš et al. (2024)	Veltliner, Riesling, Chardonnay	Still white wine	Fermentation in stainless steel or oak with bâtonnage. In stainless steel, lees removed post‐AF; in oak, contact lasted 300 days. Samples: weekly (Days 0–63), biweekly (64–150), monthly (150–300)	At 300 days, AA content was 298% higher in lees‐aged wines (∼75 mg/L) vs. 33% increase in non‐lees (∼40 mg/L). Sensory: lees‐aged wines were more robust and age‐worthy
Cerbu, Colibaba, Popîrdă, et al. (2023)	Sauvignon Blanc, Busuioacă de Bohotin	Still white and rosé wines	Twelve commercial products added to wines for 12 months lees aging + 6 months in bottle	Busuioacă: l‐Isoleucine ∼70.7 ± 25.6 mg/L; l‐asparagine ∼76.4 ± 35.3 mg/L. Sauvignon: higher l‐leucine (up to ∼96.1 ± 65.7 mg/L); l‐4‐OH‐proline lower (∼7.14 ± 3.14). Oenological products influenced AA profiles
Prokes et al. (2022)	Pinot blanc	Sparkling white (traditional)	Grapes harvested at 170/190/210 g/L sugar; fermented separately. Re‐fermentation with immobilized and conventional yeasts. Aged 24 months	Wines with conventional yeast had more AA. Higher AA in 2011 (except lysine, leucine, etc.). Higher sugar musts (210 g/L) led to more AA, except ornithine
Gnoinski, Close, et al. (2021)	Chardonnay, Pinot Noir	Sparkling white (traditional)	Yeasts treated with microwaves, ultrasound, β‐glucanase; contact for 6, 12, and 18 months	No effect early. At 18 months, β‐glucanase‐treated wines had highest AA. Proline was dominant (64%), followed by arginine (7%), asparagine (5%), and lysine (4%)
Martínez‐Lapuente et al. (2018)	Verdejo, Tempranillo	Sparkling white and rosé (traditional)	Sparkling wine aged for 9 months on lees at 11°C–13°C. Samples at 0, 3, 6, 9 months	Proline was most abundant in base wine (79% rosé, 61% white). Most AA declined to 19%–29% of initial levels after 9 months
Culbert et al. (2017)	Chardonnay, Pinot noir, Pinot Meunier, Chenin blanc, Colombard, Sauvignon blanc, Semillon	Sparkling whites (traditional, transfer, charmat) and carbonated wines	50 commercial sparkling wines analyzed for chemical composition and foam properties	Carbonated wines had significantly higher AA (esp. proline, arginine, alanine). Traditional, transfer, and charmat wines had similar AA levels (∼931–976 mg/L)
Martí‐Raga et al. (2016)	Macabeo, Xarel‐lo, Parellada and synthetic wine	Sparkling white (traditional)	Supplemented with inactive yeast or inorganic N. Aging at 16°C. Samples at 0, 1.5, 9, 18 months	Synthetic wine released 3–5 mg/L N as organic N (arginine, alanine, lysine). AA excretion began by 1.5 months. Inorganic N led to more organic N and no ammonium uptake
Martínez‐Lapuente et al. (2015)	Verdejo, Viúra, Malvasía, Albarín, Godello, Prieto Picudo, Tempranillo, Garnacha	Sparkling white and rosé (traditional)	Wines aged 9 months on lees over three vintages	All AA correlated positively with foam parameters, esp. maximum height. Nonpolar side chain AA correlated most. β‐Alanine showed highest correlation (*r* = 0.920)

*Note*: The ± indicates standard deviation.

Abbreviations: AA, amino acids; AF, alcoholic fermentation; N, nitrogen; *r*, correlation coefficient.

### 5.2 Peptides

Although peptides in wine have received less attention, they can contribute to product quality and offer health benefits (de Iseppi, Rocca, et al. 2024). According to Zhou et al. (2021), the challenges involved in isolating, characterizing, and identifying peptides in complex matrices such as wine have limited their study compared to other wine constituents. Bioactive peptides are protein fragments with beneficial effects on the human body, and their activity depends on their structure, composition, and amino acid sequence (Apud et al. 2024). These peptides typically contain fewer than 50 amino acid residues (Akbarian et al. 2022). In viable yeast cells, certain *S. cerevisiae* strains produce antimicrobial peptides such as saccharomycin—derived from the enzyme glyceraldehyde‐3‐phosphate dehydrogenase (GAPDH)—which is active against various yeasts and wine‐related bacteria (Branco et al. 2017). When yeast proteins are hydrolyzed, they can release bioactive peptides. Santos et al. (2022) identified 58 antibacterial peptide sequences from *S. cerevisiae* biomass through in silico analysis. In addition, the HSP12 protein of *S. cerevisiae* has been associated with increased sweetness perception in wine during lees autolysis, with a peptide fraction of this protein showing a correlation with the amount of lees in contact with the wine (Marchal et al. 2011, 2015).

Mannoproteins can be hydrolyzed by proteases, resulting in low‐molecular‐weight peptides, while β‐glucanases degrade the glucans bound to these mannoproteins, releasing peptides and mannans into the wine (Juega et al. 2015). During yeast autolysis, an increase in protein concentration was observed within the first 30 days of lees contact in white wines, indicating the onset of autolytic activity. In subsequent stages, these proteins were metabolized by proteases, leading to the formation of peptides and amino acids (Juega et al. 2015). In another study, after 180 days of autolysis, peptide release varied among 12 *S. cerevisiae* strains. The strain that did not multiply after 1 month of fermentation released more peptides and amino acids, while the surviving strains released less—likely due to assimilation of peptides for protein synthesis and other cellular functions. The total protein concentration initially increased, reflecting peptide release, but later declined as peptides were degraded. These findings indicate that autolysis involves both the release and progressive degradation of peptides, and that the rate of this process depends on the yeast strain used (Perpetuini et al. 2016).

Angiotensin‐converting enzyme (ACE) inhibitory activity and oxygen radical absorption capacity were evaluated in peptides released by *S. cerevisiae* during accelerated autolysis in model wine at different time points (6, 24, 48, 121, and 144 h). An increase in these bioactivities was observed up to 121 h, followed by a decrease. The hydrophobic peptide fraction was identified as the main contributor to these effects (Alcaide‐Hidalgo et al. 2007). The hydrolysis of wine lees was also shown to release peptides with antihypertensive effects via ACE inhibition, demonstrating the potential for obtaining such molecules from winemaking by‐products (Bravo et al. 2022), thus enabling the reuse of lees for human health applications. Extracts from lees derived from different yeasts (*Starmerella bacillaris*, *S. cerevisiae*, and mixed fermentations) were evaluated for bioactive peptide release following simulated gastrointestinal digestion in vitro. Results indicated that the biomass of *S. bacillaris* and *S. cerevisiae* were good sources of antihypertensive peptides, with *S. bacillaris* producing a greater number of bioactive fragments and showing anti‐obesity potential. Immunomodulatory and antimicrobial effects were observed for all yeast types, with the highest concentration of bioactive peptides found in lees from mixed fermentations (Moreira et al. 2024).

The recent study by de Iseppi, Rocca, et al. (2024) investigated the impact of autolysis on the peptide profile of synthetic wine aged on lees, where peptides originated exclusively from yeast and its autolysis. By the end of fermentation (Day 7), peptides were already detected as a result of excretion by viable yeast cells. Peptide concentration increased to approximately 1 g/L after 2 months, a value higher than that typically found in real wines due to the high yeast biomass used in the inoculum. Initially, peptide diversity was high, with 2603 sequences identified, rising to 2763 after 2 months. This was followed by a progressive decline to 2478 and 2332 sequences after 4 and 6 months, respectively. Some peptides were no longer detected at Months 4 and 6, while others emerged on Day 60, reflecting a dynamic and evolving peptide profile. Smaller and more positively charged peptides were found to be more stable, and the reduction in peptide length was attributed to yeast peptidase activity. Seven proteolytic enzymes were identified, with proteinase A and carboxypeptidase Y accounting for most of the peptide production. The origin of these peptides was traced to 314 proteins of *S. cerevisiae*, primarily from the cytosol and mitochondria. In a separate comparative study, Pegg et al. (2021) analyzed sparkling wines aged on lees for 24 and 8 months and observed a reduction in total protein abundance and enrichment of larger glycan structures on specific proteins. The study also revealed a greater abundance of yeast‐derived glycopeptides (93) compared to grape‐derived ones (11), suggesting that the majority of glycoproteins in sparkling wines are of yeast origin. These findings underscore the role of secondary fermentation and autolysis in glycopeptide formation, with potential influence on wine sensory properties.

Glutathione (GSH), a tripeptide with known antioxidant activity, is present in grapes and plays an important role in limiting browning in white wines by regenerating oxidized trans‐caffeoyltartaric acid and forming grape reaction product (GRP) (Cejudo‐Bastante et al. 2010). Its release during contact with yeast lees can enhance the antioxidant potential of the wine (Winstel et al. 2024). However, it has been observed that GSH concentration tends to decrease during lees aging (Fracassetti and Tirelli 2015) and that GSH is not released as a result of yeast autolysis (Kritzinger et al. 2013). In another study, biomass obtained from single fermentation with *S. cerevisiae* and sequential fermentation with *Hanseniaspora uvarum* followed by *S. cerevisiae* was subjected to accelerated autolysis using enzymes and high‐hydrostatic pressure (HHP), and then added to white wine. GSH release was observed in all wine samples aged on lees compared to the reference wine, suggesting a protective effect. This was particularly evident in samples containing lees from sequential fermentation treated with pressure, which showed the highest GSH content (Voce et al. 2024).

The release of GSH from wine lees has ethanol as the main factor influencing extraction efficiency (Winstel et al. 2024). Studies have shown that GSH levels in extracts from wine lees and cultivated yeast ranged from 0.15% to 0.37% (w/w), contributing to reduced oxygen consumption in model wine (de Iseppi, Curioni, et al. 2024). In addition, the use of β‐glucanase on *Saccharomyces* and non‐*Saccharomyces* yeasts increased GSH release, with a notable response from a *Hanseniaspora* strain (Voce et al. 2022). Derivatives of *S. cerevisiae* and *Torulaspora delbrueckii*, produced using acceleration technologies, showed variation in antioxidant content such as cysteine, GSH, and proteins containing cysteine residues (RPC), depending on the strain and treatment. The highest levels of GSH were found in *S. cerevisiae* subjected to HHP and in *T. delbrueckii* treated with β‐glucanase (Voce et al. 2025).

Finally, the dynamics of the antioxidant metabolome during lees aging in oak barrels were investigated, identifying 66 reactions, some of which consumed GSH, Cys, and Cys‐Gly, resulting in the formation of nucleophilic compounds rich in sulfur, nitrogen, and oxygen (Romanet et al. 2023). Antioxidant properties of these nucleophilic compounds were detected in peptides from Chardonnay base wine aged on lees in oak barrels, suggesting their role in oxidative resistance. Among the compounds identified were the amino acid l‐cysteine, GSH, and peptides such as CNS and CGGS, which may originate from yeast autolysis (Maxe et al. 2024). However, it should be noted that the protective effects of autolysis‐derived compounds are not indefinite. As demonstrated by Pons‐Mercadé et al. (2021), lees do consume oxygen, but this capacity decreases over extended aging periods (1–9 years), which may temporally limit oxidative protection in sparkling wines.

The role of peptides in contributing to kokumi—an organoleptic factor associated with flavor complexity, mouthfeel, and taste persistence—has garnered increasing attention. Although important in the sensory perception of foods and beverages, kokumi remains underexplored in the context of wine (Perenzoni et al. 2024). Among the peptides with kokumi potential, GSH is particularly noteworthy. A pioneering study by Perenzoni et al. (2024) focused on the identification of kokumi‐related oligopeptides in sparkling wines, linking this perception to interaction with the calcium‐sensing receptor (CaSR), which is essential for kokumi activation. Among the few studies addressing this topic in wine, one analyzed the kokumi peptide γ‐glutamyl‐valyl‐glycine (γ‐Glu‐Val‐Gly) in fermented beverages, including wine (Miyamura et al. 2015). This peptide is considered the most potent kokumi‐related compound (Perenzoni et al. 2024). During the production of fermented beverages, it is assumed that glutamyltranspeptidase (GGT) from malt and *S. cerevisiae* is involved in the biosynthesis of γ‐Glu‐Val‐Gly. The proteolytic activity of this enzyme has been documented in beer production, where Val‐Gly is thought to result from the degradation of barley proteins by proteases (Miyamura et al. 2015).

In the study by Perenzoni et al. (2024), commercial sparkling wines underwent lees aging for periods ranging from 36 to 130 months, and one sparkling wine was produced under controlled conditions across five vintages. Seven key dipeptides were identified, including Asp‐Leu, Asp‐Val, and Ala‐Pro, along with 35 other di‐ and tripeptides such as γ‐Glu‐Cys‐Gly (GSH) and Leu‐Ser‐Phe, all relevant to the wine's sensory profile. Among the commercial samples with varying autolysis durations, 11 peptides were identified as potential kokumi compounds, with Gly‐Val being the most prominent in sensory evaluations. Some samples also contained notable levels of glutamic acid, contributing to the wine's umami taste. The kokumi activity of Gly‐Val was more pronounced in real wines, suggesting that interactions with other compounds present only in complex wine matrices enhanced its effect. Furthermore, peptides and other fermentation‐derived compounds—such as esters, aldehydes, and higher alcohols—have been linked to wine bitterness. However, the interaction among these components and their impact on bitterness remains poorly understood, highlighting the need for further research (Luo et al. 2020).

Table 2 summarizes the diversity of research contexts related to peptides and yeast autolysis in white and sparkling wines.

**TABLE 2:** Diversity of research contexts related to peptides and yeast autolysis in white and sparkling wines.

Research topic	Summary of findings	References
Release dynamics	Initial increase in proteins within 30 days, followed by formation of peptides/amino acids	Juega et al. (2015)
Release dynamics	Peptide release after 180 days varied among 12 strains of *Saccharomyces cerevisiae*	Perpetuini et al. (2016)
Release dynamics	In synthetic wine, peptides reached ∼1 g/L and peak diversity (2763 sequences) at 2 months, then declined. Smaller, more positively charged peptides were more stable. Proteinase A and carboxypeptidase Y were the main enzymes	de Iseppi, Rocca, et al. (2024)
Bioactive activity	ACE inhibitory and antioxidant activity increased up to 121 h of autolysis, with the hydrophobic fraction being the main contributor	Alcaide‐Hidalgo et al. (2007)
Bioactive activity	Lees from different yeasts, after simulated digestion, are a source of antihypertensive, anti‐obesity, immunomodulatory, and antimicrobial peptides. Highest concentration found in mixed fermentations	Moreira et al. (2024)
Origin	Glycopeptides in sparkling wines are predominantly of yeast origin (93) vs. grape origin (11)	Pegg et al. (2021)
GSH—Release	GSH concentration decreases during aging and is not released solely by natural autolysis	Fracassetti and Tirelli (2015); Kritzinger et al. (2013)
GSH—Release	Accelerated autolysis (enzymes + high pressure) of lees promoted the release of GSH into wine	Voce et al. (2024)
GSH—Extraction factors	Ethanol is the main factor for the extraction efficiency of GSH from lees	Winstel et al. (2024)
GSH—Antioxidant effect	Lees/yeast extracts (0.15%–0.37% GSH) reduced oxygen consumption in model wine	de Iseppi, Curioni, et al. (2024)
GSH—Increased release	β‐Glucanase increased GSH release from *Saccharomyces* and non‐*Saccharomyces* yeasts	Voce et al. (2022)
GSH—Variation by strain/treatment	GSH content in yeast derivatives varied with strain and treatment; highest in *S. cerevisiae* with HHP and *T. delbrueckii* with β‐glucanase	Voce et al. (2025)
Antioxidant metabolism	Aging in oak consumed GSH, Cys, and Cys‐Gly, forming antioxidant nucleophilic compounds	Romanet et al. (2023)
Antioxidant metabolism	Identification of antioxidant peptides (CNS, CGGS, GSH) in Chardonnay wine aged in oak	Maxe et al. (2024)
Oxidative protection	The oxygen consumption capacity of lees decreases with prolonged aging (1–9 years)	Pons‐Mercadé et al. (2021)
Kokumi peptides	The peptide γ‐Glu‐Val‐Gly was identified as a potent *kokumi* compound in fermented beverages	Miyamura et al. (2015)
Kokumi peptides	11 potential *kokumi* peptides were identified in sparkling wines; Gly‐Val was the most prominent and its activity was greater in real wine matrix	Perenzoni et al. (2024)
Sensory contribution	A peptide fraction of the HSP12 protein correlated with increased sweetness perception during autolysis	Marchal et al. (2011, 2015)

*Note*: The γ symbol denotes the γ‐glutamyl bond in peptides (e.g., γ‐Glu‐Val‐Gly).

Abbreviations: ACE, angiotensin‐converting enzyme; Cys, for cysteine; Gly, glycine; GSH, glutathione; HHP, high hydrostatic pressure.

### 5.3 Polysaccharides: Mannoproteins and β‐Glucans

Among the polysaccharide macromolecules released through autolysis, mannoproteins are of particular relevance. Highly glycosylated, these glycoproteins consist predominantly of mannose (> 90%), with smaller amounts of glucose and proteins (< 10%) (Toraño et al. 2025; Martínez et al. 2019), and exhibit molecular weights ranging from 50 to 500 kDa (Dufrechou et al. 2015). The release of mannoproteins in wine is influenced by several factors, including the yeast strain or species, cell count, the physiological state of the yeast, and winemaking parameters such as grape variety, initial colloidal content of the must, temperature, agitation, and the duration of fermentation and lees aging phases (Galaz‐Torres et al. 2025). During alcoholic fermentation—particularly in the active growth phase—mannoproteins synthesized in the cytoplasm but not incorporated into the cell wall may be released, a process influenced by the initial colloid concentration in the must (Barrio‐Galán et al. 2016). The release of mannoproteins can be quantified by measuring mannose concentration after acid hydrolysis of the extracellular medium (Martínez et al. 2019). Beyond the oenological sector, mannoproteins also show promising applications in other areas of the food industry (Moreira et al. 2024; Lomolino et al. 2024).

However, mannoproteins may degrade over time and/or due to enzymatic activity, suggesting that while lees aging promotes mannoprotein release, this increase may not be sustained indefinitely. This could explain the low mannoprotein concentrations observed in sparkling wines produced by the Traditional method and aged for extended periods (≥ 6 years) (Culbert et al. 2017). In addition, lees aging of sparkling wines for 1–9 years did not result in a linear increase in polysaccharide content, as reported by Pons‐Mercadé et al. (2021). The authors attributed this to polysaccharide precipitation, which may occur due to adsorption by fining agents such as bentonite and alginate, as well as enzymatic reactions during the secondary fermentation. Similarly, Martí‐Raga et al. (2016) observed an increase in polysaccharide content up to 9 months of lees aging, but no significant difference between 9 and 18 months. In the same study, supplementation of the base wine with inactive dry yeast and inorganic nitrogen, as well as the yeast strain used in the second fermentation, significantly influenced polysaccharide concentrations. The use of a synthetic wine allowed the specific contribution of yeast to be assessed, showing an average release of 21.85 mg/L at the end of the second fermentation, increasing to 59.76 mg/L after 9 months—approximately 60% of the total polysaccharides released in real sparkling wine over the same period.

In winemaking, the addition of mannoproteins derived from the yeast cell wall is common, allowing optimization of processing technologies and expansion of the enological additive chain (Snyman et al. 2021). Current extraction methods include physicochemical techniques such as thermal extraction, sodium dodecyl sulfate treatment, US, and PEF processing, as well as enzymatic methods that break covalent bonds between glucans and mannoproteins or between glucan‐associated proteins (Gao et al. 2025). A decrease in tannin content in Chardonnay wines during lees aging has been directly associated with the increased release of mannoproteins by yeast. As shown by Martínez et al. (2019), this reduction results from the ability of mannoproteins to bind tannins, forming complexes that reduce astringency and enhance mouthfeel. This effect was observed in both PEF‐treated and untreated yeast, and was proportional to the mannoprotein concentration in the medium. In this context, lees aging in oak barrels has proven significant in modulating the chemical profile, sensory attributes, and oxidative stability of base wines. Maxe et al. (2024) found that after one year of aging in new barrels, Chardonnay base wines exhibited increased oxidative stability and changes in molecular composition, with a linear extraction of ellagitannins from the wood. However, the antioxidant metabolome varied depending on the vintage and the barrel used, highlighting the role of interactions between wood compounds, lees, and wine components.

In addition, wine colloidal stability can be improved through the use of mannoproteins, a widespread practice in the wine industry for stabilizing colloidal salts and tartrates (Millarini et al. 2020). Protein haze stability in white wines was enhanced by mannoproteins obtained via ultrafiltration of the growth medium of *Schizosaccharomyces japonicus*, which improved the thermal stability of wine proteins, reducing turbidity to approximately half its initial value in samples of Vernaccia di San Gimignano, a traditional Tuscan white wine (Millarini et al. 2020). The authors noted that mannoprotein release by *S. cerevisiae* in culture medium is limited, in contrast to the non‐*Saccharomyces* yeast used in the study. Variations in oligosaccharide composition and phosphorylation levels among strains influence surface charge and interaction area with wine macromolecules (Snyman et al. 2021). Therefore, exploring the role of non‐*Saccharomyces* yeasts in the structure of mannoproteins and their impact on the physicochemical properties of wine represents a promising area of research (Snyman et al. 2021).

β‐Glucans are polysaccharides with well‐documented bioactive properties, including immunomodulatory, antioxidant, anti‐inflammatory, antitumor, and cholesterol‐ and glucose‐lowering effects. These compounds exert their functions by activating Dectin‐1 and TLR receptors on immune cells, thereby promoting phagocytosis, cytokine production, and adaptive immune responses (Chioru et al. 2024). Structurally, β‐glucans consist of glucose molecules linked via β‐glycosidic bonds (Jofre et al. 2024) and are considered a valuable source for extraction and purification in the biotechnology industry (Varelas et al. 2016). In the food industry, β‐glucans are noted for their technological properties, functioning as thickeners, emulsifying stabilizers, and water‐retention agents (Berzosa et al. 2023). Industrially, however, they are primarily obtained from barley, oats, and mushrooms, with yeast being a less commonly used source (Dimopoulos et al. 2020). In this context, ATR‐FTIR microspectroscopy has emerged as a promising technique for selecting yeast strains based on cell wall composition, enabling the identification of strains with high macromolecule content and potential for release during autolysis (Binati et al. 2024). The activity of β‐glucanases has been widely explored regarding the release of polysaccharides associated with autolysis. Optimized enzymatic hydrolysis using these enzymes on wine yeast cell walls has shown effectiveness in protein stabilization, although still lower than that achieved with added commercial mannoproteins. This underscores the need to refine the process to balance efficacy with industrial feasibility (Moriwaki et al. 2015).

Several approaches have been proposed to investigate polysaccharide release during yeast autolysis and its possible relationship with sensory and physicochemical parameters in wine, particularly during lees aging. Lomolino et al. (2024) studied still Chardonnay wines aged on lees for 10 months, aiming to extract protein and glycoprotein fractions using different extraction protocols. These fractions were subsequently added to a carbonated model wine, with and without ethanol, to evaluate their impact on foam formation and stability. The total protein fraction reached 36.5 mg/L, the mannoprotein fraction was 1.1 mg/L, and the purified non‐mannosylated protein fraction reached 19.2 mg/L. Mannoproteins exhibited high molecular weight. Foam expansion and stability were most effective with total protein extracts, while glycoproteins and isolated proteins showed lower performance in this regard. In addition, the presence of ethanol enhanced foam expansion, although with a potential destabilizing effect.

In a study conducted by Ruipérez et al. (2022), sparkling wines made from Verdejo grapes using the traditional method were supplemented during the second fermentation with different adjuvants: β‐glucanases, authorized yeasts (containing 18%–22% polysaccharides), and yeast cell walls (containing 20%–22% soluble mannoproteins and 48%–53% polysaccharides). After 22 months of aging at 14°C–15°C, control wines showed the lowest polysaccharide concentrations, while those treated with authorized yeasts and β‐glucanases exhibited the highest levels. From a sensory perspective, wines produced with autolyzed yeasts and controls displayed profiles distinct from those made with yeast cell walls and β‐glucanases. Wines with cell wall additions stood out for their enhanced floral aromatic intensity, more intense flavor, and greater persistence. Meanwhile, those with β‐glucanases were characterized by pronounced yeast notes and increased mouthfeel volume, attributed to higher total protein content.

In addition, Pons‐Mercadé et al. (2021) evaluated white sparkling wines composed of 50% Xarel‐lo, 30% Macabeo, and 20% Parellada from nine consecutive vintages (2008–2016), stored at 12°C–15°C with lees aging periods ranging from 1 to 9 years. Lees were recovered from these wines and used in model wine experiments to simulate the autolytic process. Total polysaccharide concentrations ranged from 153 to 228 mg/L, with significant differences among vintages, although no clear trend was observed regarding aging time. Mannose analysis indicated that the released polysaccharides were predominantly mannoproteins. Furthermore, Martínez et al. (2019) used *S. cerevisiae* biomass obtained from the alcoholic fermentation of still Chardonnay wines, subjected it to PEF treatment, and reintroduced it into wines for 6 months of tank aging at 18°C with periodic lees stirring. After 30 days, treatments at 5 kV/cm yielded 230 mg/L of mannose, compared to 165 mg/L in the control. At 60 days, both PEF treatments maintained 230 mg/L, while the control only reached this concentration after 6 months. Importantly, a strong correlation was observed between mannoprotein concentration and foam‐forming capacity, regardless of the yeast treatment applied. Collectively, these studies provide valuable insights for the controlled optimization of autolysis in modern enology and open promising avenues for future research, particularly highlighting the technological potential of mannoproteins released via yeast autolysis.

Table 3 summarizes the diversity of research contexts related to polysaccharides and yeast autolysis in white wine and sparkling wines.

**TABLE 3:** Diversity of research contexts related to polysaccharides and yeast autolysis in white and sparkling wines.

Research topic	Summary of findings	Reference(s)
Release dynamics	Sparkling wines with prolonged aging (≥ 6 years) showed low concentrations of mannoproteins	Culbert et al. (2017)
Release dynamics	The polysaccharide content did not increase linearly in sparkling wines with 1–9 years of lees aging	Pons‐Mercadé et al. (2021)
Release dynamics	In sparkling wines, polysaccharides increased up to 9 months, stabilizing between 9 and 18 months. Supplementation with inactive yeast and nitrogen, and the yeast strain, influenced the levels	Martí‐Raga et al. (2016)
Release dynamics	In synthetic wine, yeast released 59.76 mg/L of polysaccharides after 9 months, representing ∼60% of the total in real sparkling wine	Martí‐Raga et al. (2016)
Stability (tannins/proteins)	The reduction of tannins in Chardonnay correlated with the release of mannoproteins during lees aging	Martínez et al. (2019)
Stability (tannins/proteins)	Mannoproteins from *Schizosaccharomyces japonicus* reduced protein haze by half in white wine	Millarini et al. (2020)
Extraction methods	PEF treatment accelerated the release of mannoproteins, which correlated with foam‐forming capacity	Martínez et al. (2019)
Sensory impact in sparkling wines	In Verdejo sparkling wines, adjuvants (cell walls, β‐glucanases) altered the sensory profile after 22 months, highlighting floral notes, persistence, mouthfeel volume, and yeasty character	Ruipérez et al. (2022)
Sensory impact in sparkling wines	Total polysaccharides (153–228 mg/L) in sparkling wines did not vary linearly with time (1–9 years), being predominantly mannoproteins	Pons‐Mercadé et al. (2021)
Yeast selection	ATR‐FTIR identified strains with high macromolecule content in the cell wall, predicting release potential	Binati et al. (2024)
Enzymatic hydrolysis	β‐Glucanases were effective in protein stabilization, but less so than the direct addition of commercial mannoproteins	Moriwaki et al. (2015)

Abbreviations: ATR‐FTIR, attenuated total reflectance–Fourier transform infrared spectroscopy; PEF, pulsed electric fields.

### 5.4 Volatile Compounds

A characteristic effect of autolysis in sparkling wines is the shift from initially floral and fruity aromas toward more complex notes, such as toasted bread, caramel, and yeast (Sawyer et al. 2021). Lees contact alters the volatile fraction and contributes to the formation of new aromatic compounds, thereby enriching the sensory profile of the final product (Martín‐Garcia, Riu‐Aumatell, et al. 2023; Juega et al. 2015). These transformations are attributed to both the release of cell‐derived compounds during autolysis and the formation of volatile compounds from aromatic precursors, catalyzed by enzymes released from dead yeast cells (Ignacia Lambert‐Royo et al. 2022). The lipids released from yeast autolysis, albeit in low concentration, are important substrates for the formation of volatile compounds (esters, ketones, aldehydes) in sparkling wines and can influence foam formation properties (Gnoinski, Close, et al. 2021). Furthermore, the sorption capacity of lees for volatile compounds is influenced by the yeast cell wall composition: mannoproteins play a key role in hydrophobic interactions, while cell wall lipids contribute to a lesser extent to the retention of lipophilic compounds (Rigou et al. 2021).

The aroma and flavor of wines result from the interaction between numerous active aromatic compounds, which can generate masking, suppression, or additive interaction effects with each other (López de Lerma et al. 2018). The perception threshold is the lowest concentration of a compound detectable by 50% of a panel. Its aromatic contribution is quantified by the odor activity value (OAV), obtained by dividing its concentration by this threshold. Compounds with similar descriptors are grouped into aromatic series, whose total value is the sum of the OAVs of their components (López de Lerma et al. 2018). The diversity of volatile compounds in sparkling wines is strongly determined by the base wine (Sawyer et al. 2021). A widely known metabolic pathway for the formation of aroma compounds, mainly in *S. cerevisiae*, is the Ehrlich pathway, which catabolizes branched‐chain and aromatic amino acids. This sequential process involves transamination to α‐keto acids, decarboxylation to aldehydes, and reduction to higher alcohols (Rumpl et al. 2025). Thus, esters, higher alcohols, and carboxylic acids are volatile compounds originating from the base wine fermentation and its refermentation (Sawyer et al. 2021). Strecker aldehydes (e.g., 2‐methylpropanal, methional) contribute to aromatic notes such as honey, malt, and floral. Their formation in wine is associated with the oxidative degradation of higher alcohols or the Strecker reaction of amino acids (Sawyer et al. 2021). Evidence suggests that some of these aldehydes may be formed early in the Ehrlich pathway, remaining masked as SO_2_ adducts and being gradually released during aging as SO_2_ is consumed (Sawyer et al. 2021).

**FIGURE 5:**
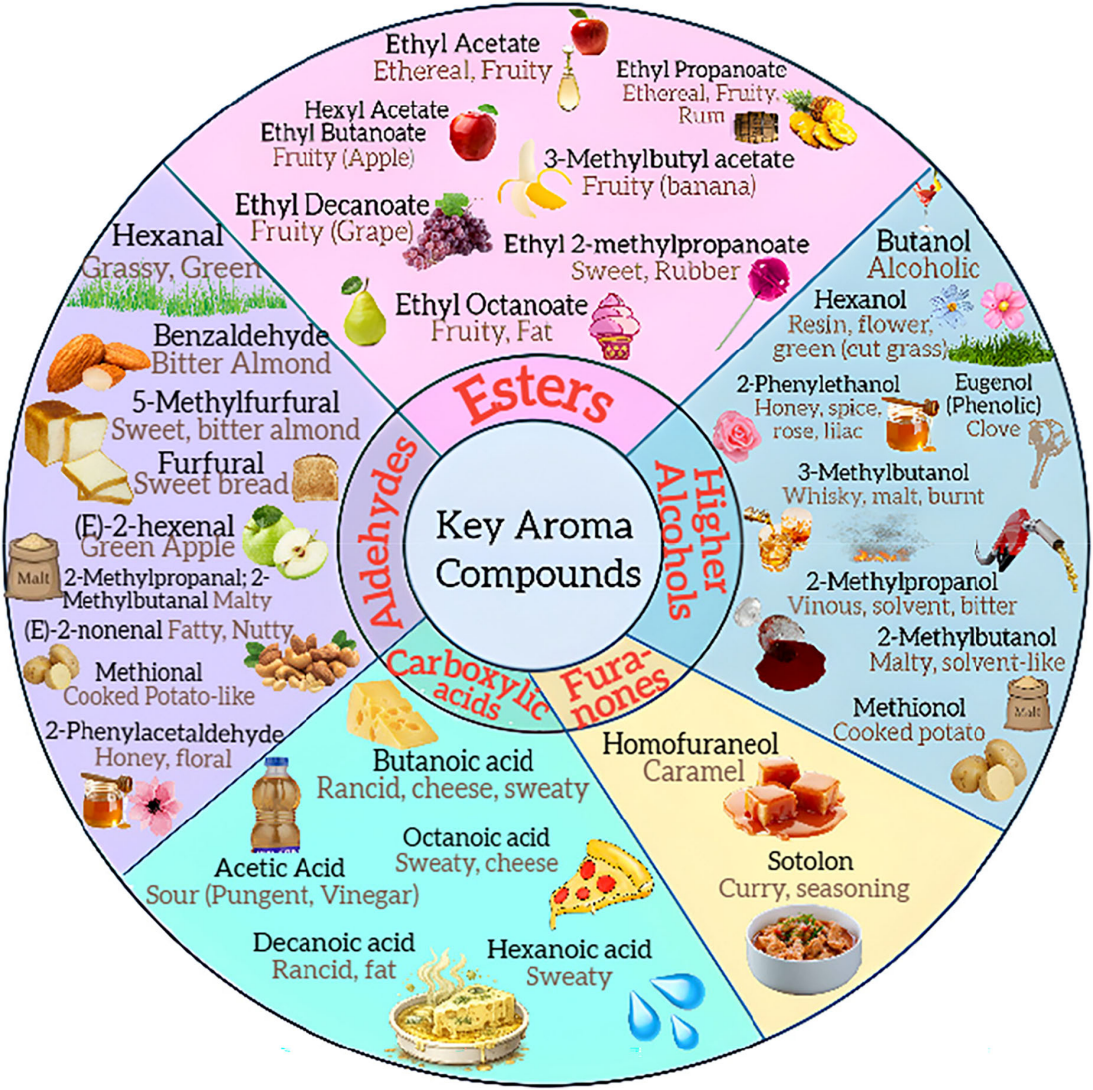
Important volatile organic compounds and their odor descriptors in sparkling wines according to the description of compounds analyzed by Sawyer et al. (2021).

Figure 5, schematized from Sawyer et al. (2021), presents examples of volatile compounds and their odor descriptors in wines. However, other aromatic groups not represented also contribute to the aroma of sparkling wines, such as norisoprenoids, including vitispiranes, identified as aging markers in sparkling wines kept on lees for up to 12 months (Ubeda et al. 2019).

For López de Lerma et al. (2018), the concentration of volatile compounds depended on the yeast strain, the form of use (free, bioimmobilized, or immobilized in alginate), or the interaction between these factors. Octanoic acid (cheese, dairy), decanoic acid (rancid, fat), and hexanol (cut grass, flower) stood out, all above 1 mg/L in sparkling wines aged for 32 months. Short‐chain fatty acids showed the highest concentrations, mainly influenced by the high content of octanoic acid, and are released into the wine during aging due to yeast lysis. Although some results depend more on the yeast strain than on the inoculation format, specific aromatic compounds were associated with the immobilization method, allowing for the classification of sparkling wines by PCA (López de Lerma et al. 2018). Ubeda et al. (2019) suggest that the fruity and floral nuances observed in aged sparkling wines may be associated with high‐impact aromatic compounds such as ethyl isobutyrate, isoamyl acetate, ethyl hexanoate, β‐phenylethanol, and diethyl succinate.

Gallardo‐Chacón et al. (2010) observed that the sorption of volatile compounds by yeast lees in sparkling wines is closely related to the hydrophobicity of the compounds. The retention of less hydrophobic volatiles decreased after the first 2 months, while the retention of more hydrophobic compounds increased up to 18 months. Chalier et al. (2007) showed that interactions between mannoproteins and aromatic compounds vary depending on the yeast strain, indicating a strain‐dependent effect. Ubeda et al. (2019) reported a decrease in ester content over time, suggesting that both sorption by lees and the thermodynamic instability of acetate esters, such as hexyl acetate, contribute to this reduction. Sawyer et al. (2021) observed changes in ethyl esters in Chardonnay and Pinot Noir wines over 24 months of aging, with an increase in esters derived from branched‐chain carboxylic acids and variations in linear‐chain esters. In addition to sorption, ester formation also occurs through chemical esterification reactions, favored by the acidic environment of wine (Sun et al. 2024). High ester production has been associated with increased autolytic activity in *S. cerevisiae* strains with varying autolytic capacities and flocculation levels (di Gianvito, Perpetuini, et al. 2018). It is worth noting that ethyl esters are primarily formed during alcoholic fermentation through reactions between alcohols and medium‐chain fatty acids (di Gianvito, Perpetuini, et al. 2018).

The high hydrophobicity and thermodynamic instability of volatile compounds such as acids and esters were also identified by Martínez‐García et al. (2021) as possible factors contributing to the reduction in total acids and ester content in sparkling wines aged on lees for 15 months. In this context, the study by la Gatta et al. (2016) adds to the discussion by showing that sparkling wines refermented with base wine lees exhibited significantly higher concentrations of esters—particularly ethyl hexanoate and ethyl octanoate, which contribute fresh fruity notes—compared to base wines without lees addition. Sun et al. (2024) found no significant changes in total ester content in white sparkling wines aged on lees, while rosé wines showed a decrease, attributed to a greater release of esterases during autolysis, which promoted ester hydrolysis. Higher alcohols increased in both wine types after 6 months of aging but declined after 8 and 10 months, likely due to oxidative reactions. la Gatta et al. (2016) emphasize the complexity of volatile compound formation in sparkling wines, which is influenced by experimental conditions, production technologies, the amount of lees present, and aging duration. Thus, the reduction in esters during aging is attributed to both the release of hydrolytic enzymes and the sorption capacity of lees, while lees lipids contribute to the formation of new esters and aldehydes (di Gianvito, Perpetuini, et al. 2018).

Ester retention by lees was observed in different Cava samples, with esters accounting for between 65% (after 40 months of autolysis) and 75% (after 16 months of aging) of the volatile fraction in the wines, while in the lees the proportion ranged from 64% (40‐month‐aged Cavas) to 82% (rosé Cava aged for 20 months). Interaction with lees significantly alters the wine's volatile profile, with a progressive increase in aging markers such as the norisoprenoids vitispirane A and 1,2‐dihydro‐1,1,6‐trimethylnaphthalene (TDN). These compounds were more abundant in both the lees and the wines subjected to 40 months of autolysis. The release of β‐glucosidases during autolysis contributes to this increase, as these enzymes cleave glycosidic bonds in nonvolatile precursors, releasing volatile terpenes and norisoprenoids.

The assimilation of volatile phenols 4‐ethylguaiacol and 4‐ethylphenol—compounds associated with sensory defects in wine, such as horse and medicinal aromas—was evaluated by Chassagne et al. (2005) using *S. cerevisiae* biomass in hydroalcoholic solutions and wine, with concentrations of 500 and 1000 µg/L of each compound. Autolyzed biomass exhibited a greater capacity for absorbing these volatile phenols compared to non‐autolyzed yeast, likely due to the release of intracellular components, including cell wall macromolecules. Among the physicochemical factors assessed, ethanol content had the most significant negative impact, as higher ethanol concentrations increase the solubility of volatiles, thereby reducing their retention by the yeast. In addition, the decrease in sorption efficiency with increasing temperature and pH was attributed to changes in polar and hydrophobic interactions between the phenols and the yeast surface, with ionization of negatively charged groups at higher pH compromising adsorption (Chassagne et al. 2005).

During the aging of sparkling wines, alcohols and acids remained relatively stable, showing no significant variations (Ubeda et al. 2019). The furanic compound furfural, formed through sugar degradation, appears after the second fermentation and its concentration doubles after 12 months of lees contact. While most esters either decrease or remain stable, certain compounds—such as ethyl lactate, methyl 2‐oxononanoate, and diethyl succinate—increase over time. Ethyl lactate and diethyl succinate have been identified as aging markers (Ubeda et al. 2019), and the increase in diethyl succinate during lees aging was also confirmed by Martínez‐García et al. (2021). Additional aging markers for Cava sparkling wines include isoamyl and hexyl acetates, ethyl decanoate, and vitispiranes for young wines (under 9 months), and vitispiranes and TDN for more mature wines (over 20 months). Diethyl succinate was consistently observed as a marker throughout the aging process (Francioli et al. 2003). In Croatian commercial sparkling wines, the presence of diethyl succinate and diethyl glutarate was also associated with aging, with their concentrations influenced either by yeast autolysis or by chemical esterification reactions occurring during maturation (Jagatić Korenika et al. 2020).

The study by Charnock et al. (2023) evaluated the impact of the Maillard reaction in sparkling wines, focusing on sugar dosage in the *liqueur d'expédition* after yeast removal at the end of autolysis. It was found that the duration of aging had a greater effect on the chemical composition of the wines than the sugar composition of the dosage. Alanine and glycine were the amino acids most responsible for variability among the samples, showing a decrease over time, suggesting a possible link to Maillard reaction activity. Compounds such as benzaldehyde and ethyl 3‐mercaptopropionate varied significantly between wines aged for 0 and 18 months. In an earlier study, Charnock et al. (2022) discussed the Maillard reaction in traditional method sparkling wines, emphasizing that, due to low temperatures (15 ± 3°C) and low pH (3–4), the reaction does not typically progress beyond intermediate stages. Nevertheless, the formation of furanic compounds such as 5‐hydroxymethylfurfural (5‐HMF) was identified as a potential aging marker linked to Maillard reactions. In addition, metal ions in wine may catalyze the reaction by acting as Lewis acids.

The production of yeast lysates through accelerated autolysis, enzymatic hydrolysis with Alcalase, and mechanical lysis—followed by their addition during the fermentation of Chardonnay white wines—was evaluated with a focus on volatile profile modulation (Onetto et al. 2024). Fermentation with 10% (v/v) lees lysate significantly increased the concentration of volatile esters, with lysates treated with Alcalase having the most pronounced effect, as they released more amino acids and, consequently, a greater number of volatile compounds were identified. The use of white wine lees as a growth enhancer for lactic acid bacteria during malolactic fermentation in red wines was also explored (Balmaseda et al. 2024). The addition of lees significantly increased the ester concentration compared to wines without this supplementation. In Merlot wines, compounds such as ethyl 2‐methylpropanoate and ethyl 3‐methylbutanoate stood out, while in Petit Verdot, an increase in 2‐methylpropyl acetate was observed. This enhancement was attributed to the greater nitrogen availability from the lees. Studies indicate that after malolactic fermentation, levels of short‐ and branched‐chain fatty acids tend to rise in wines with higher nitrogen content (Balmaseda et al. 2024). In addition, nitrogen supplementation can boost the formation of medium‐chain fatty acid esters, due to increased fatty acid synthesis (Onetto et al. 2024). These findings highlight the technological potential of yeast biomass derived from autolysis, supporting its reuse in the wine industry to enhance aromatic quality and valorize fermentation by‐products.

To complement the discussion on the aromatic modulation promoted by autolysis in sparkling and still wines, Table 4 provides a structured overview of key studies published from 2015 to 2025. The selected works encompass a broad diversity of grape varieties, wine styles, and methodological approaches used to investigate the volatile fraction during or after yeast autolysis. Experimental parameters such as yeast strain selection, autolysis acceleration techniques, fermentation regimes, and storage conditions are detailed to highlight their influence on the production or retention of volatile compounds. Analytical methods (especially gas chromatography coupled with mass spectrometry) and sensory evaluation techniques are also reported to illustrate how volatile composition translates into perceptible sensory effects.

**TABLE 4:** Summary of studies from the last decade (2015–2025) on the impact of autolysis on the volatile fraction of wines. The table includes information on grape varieties, wine type, autolysis conditions, chromatographic and sensory methods, and main findings related to aroma.

References (years)	Grape variety	Wine type and conditions	Autolysis conditions and experimental parameters	Chromatography and sensory methods	Main volatile outcomes
Voce et al. (2024)	Sauvignon Blanc, Pinot Gris	Still white wine	5 % (v/v) lysate from *Hanseniaspora uvarum* and *Saccharomyces cerevisiae*, 3 months at 20°C, biweekly bâtonnage	SPME‑GC‑MS; sensory difference test (attribute intensity)	Increased complexity and intensity: higher acetic esters, higher alcohols, volatile phenols (e.g., ethyl acetate, 2‐/3‑methyl‑1‑butanol, 4‑ethylguaiacol). Lower aldehydes in treated
Sun et al. (2024)	Chardonnay, Pinot Noir	Sparkling white and rosé (traditional method)	Aging on lees for 3, 6, 8, 10 months at 15°C–18°C	GC–MS; descriptive sensory with modified frequency‑intensity approach	White: ethyl octanoate (OAV ≈ 3.2); Rosé: total esters ↓22 % at 10 months. Isoamyl alcohol increased until Month 6 then ↓. TDN appeared after 8 months
Martín‐Garcia, Abarca‐Rivas, et al. (2023)	—	Sparkling wine (Cava, traditional method)	Aging on lees at 16°C; sampling at 0, 4, 8, 12, 21 months after ≥ 9 months initial contact	HS‑SPME‑GC‑MS	> 60 volatile compounds identified; furans dominated aging profile, independent of lees contact
Martín‐García, Riu‐Aumatell, et al. (2023)	Macabeu, Xarel‑lo, Parellada; Garnatxa, Trepat	Sparkling white and rosé wines (traditional method)	Cava samples aged 16, 20, and 40 months; lees frozen, lyophilized	HS‑SPME‑GC‑MS	68 compounds identified, 19 exclusive to lees. Longer aged Cavas show higher volatile diversity and higher wine‑lees similarity (up to 53% at 40 months)
Santos et al. (2023)	Antão Vaz, Rabo de Ovelha, Viosinho, Fernão Pires, Arinto, Verdelho, Manteúdo, Gouveio, Semillon, Perrum, and Diagalves	Still white wines (varietal and blend); 0–120 mg/L SO_2_; aged on lees 3 months + bottle aging to 12 months	Contact with fine lees 3 months at 16°C, then bottled and stored up to 12 months	HS‑SPME‑GC‑MS	30/60 mg/L SO_2_ wines influenced by isoamyl decanoate; high SO_2_ protected esters (isoamyl acetate). At 12 months, succinate and decanoic acid dominant; hexanoate at 120 mg/L
Stamenković Stojanović et al. (2023)	Grašac	Still white wine (sequential with *H. uvarum* + *S. cerevisiae*)	6 months with commercial yeast derivatives (20 or 40 g/hL), mixed twice per 3 days agitation	HS‐SPME‑GC‑MS; descriptive sensory panel	All volatile compounds within acceptable limits. Derivatives retained fruity/floral attributes; higher dosage enhanced floral profile
Cerbu, Colibaba, Luchian, et al. (2023)	Sauvignon blanc, Busuioaca de Bohotin	Still white and rosé wines (commercial mixes)	Twelve commercial lees products used 12 months on lees + 6 months in bottle	Descriptive sensory (olfactory/gustatory descriptors, 1–9 scale)	Mature aging reduced vegetal/mineral notes, amplified bread/biscuit odors. Exotic fruit aromas evident, dose‑dependent intensification with no linear pattern
Ignacia Lambert‐Royo et al. (2022)	Chardonnay	Sparkling wine (traditional method)	Additions of dry yeast lysate, protein extract or *Torulaspora delbrueckii*; aging 3–18 months	HS‑SPME‑GC‑MS; descriptive sensory (visual, olfactory, gustatory)	For < 9 months, *T. delbrueckii* and protein extract preserved fruity volatiles. At 18 months, *T. delbrueckii* yielded the most enduring fruit notes
Sawyer et al. (2021)	Chardonnay, Pinot Noir	Sparkling wines (traditional method), aging 6, 12, 24 months	Lees contact 15°C for 6, 12, 24 months; base wine aged with/without lees	HS‑SPME‑GC‑MS; panel sensory (orthonasal and retronasal)	Autolysis products less impactful aromatically than expected. Wine base composition dominated early aromatics within first 24 months
Mislata et al. (2021)	Chardonnay, Xarel‐lo	Sparkling wine (traditional method)	Primary fermentations with 5 yeast strains (3—*S. cerevisiae*, 1—*Metschnikowia pulcherrima*, 1—*T. delbrueckii*), sparkling aging 18 months	SPME‑GC; quantitative descriptive sensory trained panel	*T. delbrueckii* maintained higher aroma levels at 18 months compared to *S. cerevisiae*, producing more complex sensory profiles
Martínez‐García et al. (2021)	Pedro Ximenez, Moscatel	Sparkling wine (traditional method)	Two *S. cerevisiae* strains; aging 15 months with samples at 3, 6, 9, 12, 15 months	GC‐FID and SBSE‑GC‑MS; triangular and descriptive sensory tests	Aging had greater impact than strain. Native yeast wines scored higher aromas at all aging times per PCA and sensory tests
Jagatić Korenika et al. (2020)	Multiple varieties (Chardonnay, Pinot noir, Portugizer, Kraljevina, Manzoni bianco)	Sparkling wine (traditional method) from three regions	Commercial Croatian Cavas, various autolysis periods	SPE‑GC‑MS; OAV and ROC odor contribution analysis	Region and lees contact affected esters and higher alcohols. Esters with OAV > 1 were key; succinate/glutarate diethyl markers of autolysis prominent
Ubeda et al. (2019)	Local (Spain)	Sparkling wine (traditional method), aging 12 months	14 ± 2°C for 12 months with analyses at 0, 3, 6, 9, 12 months	HS‑SPME‑GC‑MS; GC‑Olfactometry; descriptive sensory panel	Ester loss over time, increased norisoprenoides; aged wines (9–12 months) had stronger bread, yeast, toast aroma despite sensory floral decrease
di Gianvito, Perpetuini, et al. (2018)	—	Sparkling wine (traditional method), sampled at intervals up to 180 days	Six *S. cerevisiae* strains with varying flocculation/autolysis, sampling 7–180 days	GC‑MS‑SPME	Flocculation/autolysis phenotype affected aromatic release; esters varied by strain. Flocculant strains produced higher alcohols and esters early on
la Gatta et al. (2016)	Bombino	Sparkling wine (traditional method), varying lees dose added	0, 30, 60 mL lees per L; 18 months aging	HS‑SPME‑GC‑MS; descriptive sensory (olfactory and gustatory)	Higher lees dose increased volatile esters and fatty acids; improved aroma persistence; wines with lees had stronger green fruit/leaven notes; odor intensity higher in no‑lees
Pérez‐Magariño et al. (2015)	Verdejo, Godello, Tempranillo, Garnacha	Sparkling white and rosé wines (traditional method) 9 months aging	Two vintages; with/without commercial yeast lysates; aging 0–9 months at 11°C–13°C	GC–MS; descriptive sensory (7‐point scale)	Variety and aging time strongly affected volatile patterns. Increased branched‐chain esters, ethyl lactate, 2‑phenylethanol; decreased linear‐chain esters. Fruit aroma enhanced by manoprotein‐rich additives

Abbreviations: FID, flame ionization detector; GC–MS, gas chromatography–mass spectrometry; GC‐O, gas chromatography–olfactometry; HHP, high hydrostatic pressure; HS‐SPME, headspace solid phase microextraction; OAV, odor activity value; PCA, principal component analysis; ROC, relative odor contribution; SBSE, stir bar sorptive extraction; SO_2_, sulfur dioxide; TDN, 1,1,6‐trimethyl‐1,2‐dihydronaphthalene.

## 6 Emerging Technologies for Autolysis Acceleration

**FIGURE 6:**
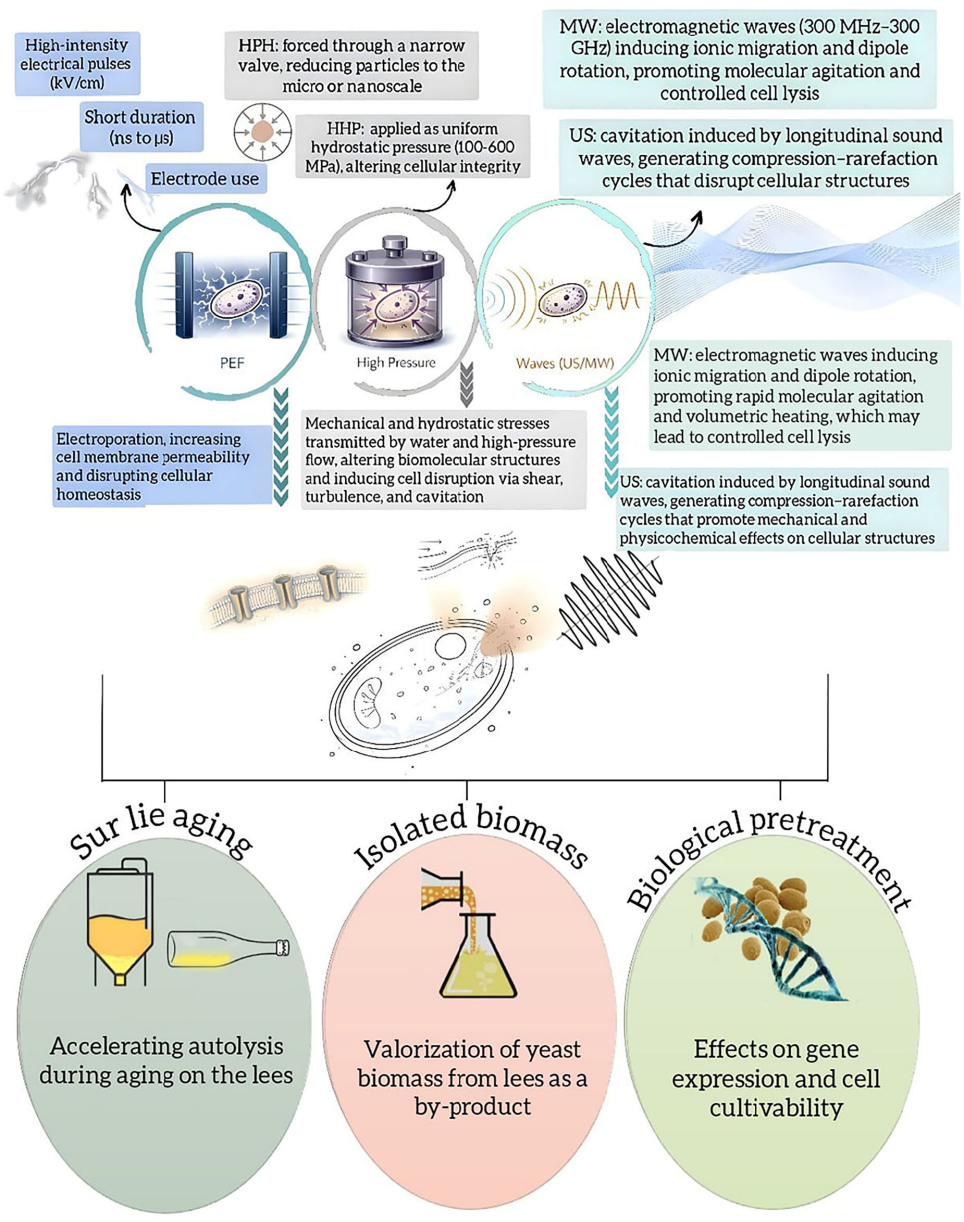
Modes of operation of pulsed electric fields (PEF), high hydrostatic pressure (HHP), high‐pressure homogenization (HPH), ultrasound (US), and microwaves (MW), the cellular damage they cause to yeasts, and their main potential applications for oenological yeasts.

Recent studies have highlighted the application of emerging technologies to accelerate yeast autolysis in winemaking, including PEFs, HHP, HPH, US, and MWs. The International Organisation of Vine and Wine (OIV) has authorized the use of US and high‐intensity electric pulses to enhance the extraction of grape compounds and reduce maceration time, as well as the application of high pressure to inactivate microorganisms and decrease the need for sulfur dioxide (Voce et al. 2024; OIV 2022). However, these technologies also show potential to induce autolysis, enhancing lees aging (Voce et al. 2024). As a result, recent research has focused on optimizing autolysis in wines and exploring the reuse of lees for alternative applications. Figure 6 summarizes these technologies, their mode of operation, the cellular damage caused to yeasts, and their main potential applications for oenological yeasts.

### 6.1 PEF

PEF treatment involves the application of high‐intensity electrical pulses (kV/cm) of short duration (ns to µs) to a product placed between two electrodes (Raso 2023; Feng et al. 2022). This process induces electroporation, increasing cell membrane permeability and disrupting homeostasis, which can lead to microbial cell death (Delso et al. 2023; Martínez et al. 2019). Although microorganisms damaged by PEF can recover under optimal conditions (Martínez et al. 2019), the acidic pH and ethanol content of wine inhibit this regeneration, promoting cell death and accelerating autolysis. After treatment, extraction conditions such as pH, temperature, and duration are critical for optimizing the release of compounds of interest, as demonstrated with GSH in *S. cerevisiae* (Berzosa et al. 2024).

Among the various applications of PEF in winemaking are the acceleration of maceration, enhanced extraction of functional compounds, improvement in wine color, inactivation of undesirable microorganisms, and promotion of aromatic compound formation (Feng et al. 2022). Unlike thermal processes such as thermolysis, PEF does not cause heat damage, thereby preventing the formation of odor‐active compounds typically associated with excessive heating (Martínez et al. 2019). However, research on using PEF to accelerate autolysis in wines remains limited, with most studies focusing on red wines.

PEF treatments at intensities of 12, 15, and 18 kV/cm with 3‐µs pulses were effective in releasing GSH from *S. cerevisiae* cells grown in culture media, regardless of pH (4, 6, or 8) and temperature (4°C or 25°C). Membrane permeability increased with field intensity, reaching about 35% permeabilization after 20 µs at 12 kV/cm, and over 90% permeability between 50 and 150 µs (Berzosa et al. 2024). In a separate study, Okamoto et al. (2021) applied 20 kV/cm with 0.6‐µs pulses, causing membrane damage while leaving the cell wall intact; subsequent wall collapse during incubation facilitated the release of autolytic enzymes. Moreover, mannoprotein release in wine was assessed using lees from *S. cerevisiae* derived from white wine and treated with 15 kV/cm, using 25 pulses of 3 µs. These lees were added to a red wine and aged at 18°C for 3 months. The treatment achieved approximately 80% mannoprotein release in just 3 weeks, whereas the control required about 3 months to reach comparable levels. Enzymatic assays showed that PEF significantly accelerated the release of β‑glucanase and protease (Maza et al. 2020).

In Chardonnay wines, PEF treatments ranging from 5 to 25 kV/cm increased mannoprotein concentrations by 40% and 60% in samples treated at 5 and 10 kV/cm, respectively, after 7 days. The highest concentrations were reached within 30 days in the treated samples, whereas the control group required 6 months to achieve similar levels (Martínez et al. 2019). The authors concluded that PEF effectively accelerates lees aging and mitigates its associated drawbacks, even at low intensities, enabling the use of cost‐effective equipment by wineries. Furthermore, applying PEF to industrial *S. cerevisiae* lees enhanced mannoprotein release in a manner dependent on treatment intensity and duration, without altering their functional properties (Martínez et al. 2019). Regarding the amino acid fraction, in the context of accelerating autolysis in yeast lysates, Yang et al. (2021) reported that treatments at 7 kV/cm led to a 149.36% increase in total amino acid content, particularly aspartic and glutamic acids, which are important for umami flavor perception. These findings suggest promising applications of PEF in the autolysis of *S. cerevisiae* for winemaking. Table 5 summarizes the research that applied PEF in the context of wine yeast autolysis.

**TABLE 5:** Research that applied PEF in the context of wine yeast autolysis.

PEF conditions	Yeast/matrix	Target compounds	Main outcomes	Wine type/context	References
12–18 kV/cm, 3 µs pulses (20–150 µs total)	*Saccharomyces cerevisiae* (culture media)	Glutathione	Up to > 90% membrane permeabilization; efficient glutathione release regardless of pH (4–8) and temperature (4°C–25°C)	Model system	Berzosa et al. (2024)
20 kV/cm, 0.6 µs pulses	*S. cerevisiae* (culture media)	Autolytic enzymes	Facilitated release of intracellular autolytic enzymes during incubation	Model system	Okamoto et al. (2021)
15 kV/cm, 25 pulses of 3 µs	*S. cerevisiae* lees (white wine origin)	Mannoproteins, β‐glucanase, protease	∼80% mannoprotein release in 3 weeks vs. 3 months in control	Lees added to red wine	Maza et al. (2020)
5–25 kV/cm, pulses 30–105 µs	*S. cerevisiae* lees	Mannoproteins	40%–60% increase after 7 days; target levels reached in 30 days vs. 6 months (control)	Chardonnay wine	Martínez et al. (2019)
3–7 kV/cm, 100 µs pulses	*S. cerevisiae* lysates	Amino acids (Asp, Glu)	149.36% increase in total amino acids; enhancement of umami‐related compounds	Yeast lysates	Yang et al. (2021)

Abbreviations: Asp, aspartic acid; Glu, glutamic acid; PEF, pulsed electric fields.

### 6.2 HHP and HPH

HHP delivers high pressures (100–600 MPa) using water as the transmitting medium, thereby avoiding thermal damage to the product. In contrast, HPH induces yeast cell disruption through cavitation, shear forces, and turbulence by forcing the sample through a narrow valve. Although these technologies are predominantly used for microbial stabilization in must and wine, their application to accelerate yeast autolysis in winemaking remains underexplored (Blanco‐Huerta et al. 2024). In a recent study, *S. cerevisiae* was treated at 400, 500, and 600 MPa for 3, 5, and 10 min in a model wine system, followed by 42 days of lees aging. HHP treatment led to reduced release of nucleic acids, proteins, and polysaccharides compared to the control, suggesting enzymatic denaturation limited cellular autolysis, particularly at 600 MPa (Blanco‐Huerta et al. 2024). In contrast, Voce et al. (2024) showed that HHP treatment at 400 MPa for 8 min at 30°C increased polysaccharide release, indicating that HHP parameters may be optimized to enhance autolysis. The authors further highlight that implementing sequential fermentation with *H. uvarum* and *S. cerevisiae*, followed by HHP treatment and lees aging, improves color development and enhances antioxidant protection, underscoring the potential of integrating biotechnological and physical treatments.

Furthermore, Voce et al. (2025) explored the combined use of US and HHP (400 MPa, 8 min, 30°C) to induce autolysis in *S. cerevisiae* and *T. delbrueckii*. Following the addition of yeast derivatives to a white wine, chemical characterization at 2 and 6 months showed that *T. delbrueckii* is a promising candidate for producing yeast derivatives, exhibiting performance comparable to *S. cerevisiae*. These results reinforce the potential of emerging technologies such as US and HHP to complement traditional thermal and enzymatic approaches in yeast derivative production. The use of HPH as a pretreatment to induce autolysis in *S. cerevisiae* was investigated by Dimopoulos et al. (2020), focusing on yeast extract production and β‐glucan extraction. The degree of yeast cell disintegration was influenced by both pressure intensity and the number of passes through the homogenizer. At 100 bar, no significant effect was observed after eight passes, whereas higher pressures led to increased cell disruption. In addition, the β‐glucan content in the solid residue rose with treatment intensity and autolysis duration, while protein content declined due to cell rupture, suggesting the potential of HPH for yeast biomass valorization.

As discussed in Section 4 of this review, HPH has also been studied in terms of its effects on the autolytic process and gene expression in *S. cerevisiae* (Gottardi et al. 2022). The application of sublethal pressure (100 MPa) induced genetic reprogramming, altering yeast metabolism and enhancing the production of alcohols that positively affected the volatilome and sensory properties within 48 h. The HPH pretreatment induced a state of autolysis, reducing the production of ketones and alcohols after 2 h. However, after 48 h, there was an increase in the release of compounds such as benzaldehyde, ethanol, and ethyl esters, which may influence the sensory quality of sparkling wines. Finally, studies by Comuzzo et al. (2015) showed that HPH treatments at 50, 100, and 150 MPa did not fully inactivate *S. bayanus* cells, unlike thermolysis, which achieved complete inactivation. HPH induced autolysis in *S. bayanus*, releasing macromolecules under wine‐like conditions. At 150 MPa, the treatment was most effective, releasing compounds comparable to those of thermal autolysis and accelerating *sur lie* aging. In addition, HPH led to a lower concentration of free fatty acids and a higher presence of ethyl esters, which are beneficial for winemaking. These findings highlight HHP and HPH as promising technologies for accelerating autolysis, enhancing compound release, and optimizing yeast‐derived product development, with substantial potential for innovation in oenology.

Although some studies report divergent results regarding the effectiveness of HHP and HPH in releasing compounds such as polysaccharides and proteins, most research suggests that these technologies hold potential to accelerate autolysis and improve wine quality. The observed variations can be attributed to differences in experimental conditions, analytical methods, and specific interactions between yeast strains and the medium. These factors highlight the need for further investigation to optimize these technologies for specific applications in winemaking. Table 6 summarizes the research that applied HHP and HPH in the context of wine yeast autolysis.

**TABLE 6:** Research that applied HHP and HPH in the context of wine yeast autolysis.

Technology	Processing conditions	Yeast/matrix	Target compounds	Main outcomes	Application/context	References
HHP	400–600 MPa, 3–10 min	*Saccharomyces cerevisiae* (model wine, lees aging 42 days)	Nucleic acids, proteins, polysaccharides	Reduced compound release at ≥ 600 MPa, suggesting limited autolysis due to enzyme denaturation	Model wine system	Blanco‐Huerta et al. (2024)
HHP	400 MPa, 8 min, 30°C	*Hanseniaspora uvarum*; *S. cerevisiae*	Polysaccharides, antioxidants	Improved color development and enhanced antioxidant protection during lees aging	Model wine for lees production; treated lees added to real white wine for aging	Voce et al. (2024)
HHP + ultrasound	US + 400 MPa, 8 min, 30°C	*S. cerevisiae*; *Torulaspora delbrueckii*	Yeast‐derived compounds	*T. delbrueckii* showed performance comparable to *S. cerevisiae* in white wine	Treatments applied to yeast lees, followed by addition to white wine for aging	Voce et al. (2025)
HPH	50–150 MPa, multiple passes	*Saccharomyces bayanus* (wine‐like conditions)	Macromolecules, ethyl esters	Accelerated autolysis; 150 MPa most effective; lower free fatty acids, higher ethyl esters	*Sur lie* aging	Comuzzo et al. (2015)
HPH	≥ 100 bar, up to 8 passes	*S. cerevisiae*	β‐Glucans, proteins	Increased β‐glucan content in solid residue; protein loss due to rupture	Yeast biomass valorization	Dimopoulos et al. (2020)
HPH	100 MPa	*S. cerevisiae*	Alcohols, esters, ketones	Enhanced volatilome after 48 h; increased benzaldehyde, ethanol, and ethyl esters	Sparkling wine context	Gottardi et al. (2022)

Abbreviations: HHP, high hydrostatic pressure; HPH, high pressure homogenization; US: ultrasound.

### 6.3 US and MW

US in liquid systems relies on cavitation created by longitudinal waves, generating alternating zones of compression and rarefaction. This allows for scalability and reduced energy consumption depending on the matrix and desired outcome. In contrast, MWs work at the molecular level via ionic migration and dipole rotation, using electromagnetic waves in the range of 300 MHz to 300 GHz with wavelengths from 1 m to 1 mm. In sparkling wines (Chardonnay‑Pinot Noir), MW treatments (1100 W, 99°C), and US (50 kHz, 350 W) were applied to lyse *S. cerevisiae* in the tirage liquor, and samples were evaluated after 6, 12, and 18 months. Total protein remained stable (∼80 mg/L after 12 months), while free amino acids varied with treatment and bottle age. After 18 months, β‑glucanase treatment increased amino acid concentration versus control and US treatment, with proline accounting for 64% of the total. Total lipids increased significantly with β‑glucanase and US (phospholipids and monoacylglycerols) or MW (triacylglycerols). Snyman et al. (2021) reported a synergistic effect between US and β‑1,3‑glucanase, maximizing carbohydrate and protein release after 20 h of incubation. Lees from sparkling wine subjected to acid–base extraction and US‐assisted autolysis (25–45 kHz) showed that autolysis was superior regardless of US frequency, with higher yields of β‑glucans. This suggests that lees composition and processing conditions significantly influence the outcome.

Studies on red wines indicate comparable effects: Muñoz García et al. (2023) demonstrated that US and MW enhance the extraction of amino acids, polysaccharides, and manoproteins from lees in red wines, particularly when combined with inactive yeast. However, both treatments significantly reduced color, anthocyanins, and tannins. US also reduced key volatile aroma compounds—negatively affecting red and floral sensory notes—while MW better preserved the sensory profile, with reduced astringency compared to the control. Del Fresno et al. (2018) showed that US applied during aging of red wines with lees and oak chips decreased anthocyanin and volatile compound levels compared to controls, while increasing polysaccharide content. Total phenols and volatile acidity remained unaffected.

As with other emerging technologies, US and MW affect the physicochemical and sensory characteristics of wine by releasing intracellular compounds via yeast cell degradation. However, results vary depending on processing parameters. For a winery‐scale application, it is recommended to run preliminary small‐batch trials to adjust and evaluate the feasibility of implementing these techniques at the production scale.

## 7 Conclusions

This review underscores recent advances in the study of yeast autolysis in winemaking, focusing on white and sparkling wines. *S. cerevisiae* remains the central species, while interest in non‐*Saccharomyces* yeasts continues to grow. The predominance of research on sparkling wines produced by the Traditional Method underscores a gap in knowledge regarding other wine categories, such as Charmat‐method sparkling wines and long‐macerated white wines, where autolysis may also influence physicochemical and sensory attributes. Autolysis durations typically range from 6 to 18 months, with some cases extending up to 9 years. The most common temperatures for conducting autolysis are between 12°C and 18°C. Synthetic wine models have proven useful in isolating yeast effects, though they fall short of replicating the complexity of real wine matrices. The volatile fraction resulting from autolysis is receiving increasing attention, although data remain inconsistent. Diethyl succinate stands out as the main volatile marker. Emerging technologies such as PEFs, HHP, HPH, US, and MWs applications show promise for accelerating autolysis and enhancing the release of oenologically relevant compounds. Moreover, the identification of autophagy‐related genes and the use of techniques such as ATR‐FTIR and NMR contribute to the targeted selection of autolytic yeast strains. In addition, the sensory potential of kokumi peptides derived from autolysis warrants further exploration. Ultimately, expanding studies to minimally processed wines is essential, as autolysis may persist after bottling and influence the final product in ways not yet fully understood.

## Author Contributions

**José Ricardo Machado dos Santos**: conceptualization, investigation, writing – original draft, methodology, formal analysis, visualization, data curation. **Aniela Pinto Kempka**: conceptualization, investigation, funding acquisition, validation, writing – review and editing, supervision, resources, project administration.

## Funding

This work was supported in part by the Fundação de Amparo à Pesquisa e Inovação do Estado de Santa Catarina (FAPESC—Termo de outorga 2024TR002214). Aniela Pinto Kempka is granted a fellowship (PQ2) from CNPq—Brazil (303915/2022‐6).

## Conflicts of Interest

The authors declare no conflicts of interest.
